# Mitochondria protective and anti-apoptotic effects of peripheral benzodiazepine receptor and its ligands on the treatment of asthma in vitro and vivo

**DOI:** 10.1186/s12950-024-00383-0

**Published:** 2024-04-19

**Authors:** Yurui Liu, Zhengze Zhang, Yuewen He, Ruogen Li, Yuhao Zhang, Hao Liu, Yong Wang, Wuhua Ma

**Affiliations:** 1 Guangzhou University of Chinese Medicine, Guangzhou, P.R. China; 2 Department of Anesthesiology, The First Affiliated Hospital of Guangzhou University of Chinese Medicine, 12 Jichang Road, 510405 Guangzhou, P.R. China

**Keywords:** Asthma, Bronchial epithelial cells, Peripheral benzodiazepine receptors, Mitochondrial permeability transition pore, Apoptosis

## Abstract

**Background:**

Asthma is a prevalent respiratory inflammatory disease. Abnormal apoptosis of bronchial epithelial cells is one of the major factors in the progression of asthma. Peripheral benzodiazepine receptors are highly expressed in bronchial epithelial cells, which act as a component of the mitochondrial permeability transition pore to regulate its opening and closing and apoptosis of bronchial epithelial cells. We aimed to investigate the mechanisms by which peripheral benzodiazepine receptor and its ligands, agonist 4’-Chlorodiazepam (Ro5-4864) and antagonist 1-(2-chlorophenyl)-N-methyl-N-(1-methylpropyl)-3-isoquinolinecarboxamide (PK 11,195), modulate the mitochondrial function and cell apoptosis in the treatment of asthma.

**Methods:**

In vitro study, Ro5-4864 and PK 11,195 were utilized to pretreat cells prior to the inflammatory injury induced by Lipopolysaccharide. The reactive oxygen species, the apoptosis of cell, the mitochondrial membrane potentials, the ultrastructures of the mitochondria and the expression levels of peripheral benzodiazepine receptors and apoptosis-related proteins and genes were detected. In vivo study, mice were administrated intraperitoneally with Ro5-4864 and PK 11,195 before sensitized and challenged by ovalbumin. Serum IgE and bronchoalveolar lavage fluid cytokines were detected, and lung tissues were underwent the histopathological examination.

**Results:**

The ligands of peripheral benzodiazepine receptor counteracted the effects of the increase of reactive oxygen species, the elevated extent of apoptosis, the decrease of mitochondrial membrane potentials and the disruption of mitochondrial ultrastructures induced by Lipopolysaccharide. The ligands also promoted the expression of anti-apoptosis-related proteins and genes and inhibited the expression of pro-apoptosis-related proteins and genes. Besides, the ligands reduced the levels of serum IgE and bronchoalveolar lavage fluid cytokines in asthmatic mice and attenuated the histopathological damage of lungs.

**Conclusion:**

Peripheral benzodiazepine receptor serves as a potential therapeutic target for the treatment of asthma, with its ligands exerting mitochondrial protective and anti-apoptotic effects on bronchial epithelial cells.

**Supplementary Information:**

The online version contains supplementary material available at 10.1186/s12950-024-00383-0.

## Background

Asthma is a common chronic respiratory inflammatory disease with symptoms including wheezing, chest tightness, dyspnea and coughing, and its pathophysiology is characterized by the airway hyperresponsiveness (AHR) and reversible airflow obstruction resulted from the airway inflammation, mucus secretion and airway remodeling [1]. According to the Global Strategy for Asthma Management and Prevention updated by the Global Initiative for Asthma in 2023, the global morbidity and prevalence of asthma is increasing [2], which has become a severe health issue worldwide. With the appropriate treatments, the conditions of the sufferers can be controlled. However, there exist some patients whose symptoms can not be controlled despite receiving conventional therapies including inhaled glucocorticoids and β2-agonists, which is defined as refractory asthma [3]. The side effects of high dose glucocorticoids make the therapeutic process more difficult, and the application of targeting drugs such as monoclonal antibodies has been constrained due to their uncertain effectiveness and high cost [4]. Based on above, it is of great clinical significance to seek the new potential therapeutic target for asthma and to unravel its mechanism of action.

Bronchial epithelial cells (BECs), the first barriers against asthma allergens [5], possess the functions of the physical barrier, the cilia removal and the innate immune response [6]. However, BECs are considered as more than just the structural barriers. They empress pattern recognition receptors that detect the stimuli and secrete “alarmin cytokines” including IL-25, IL-33 and TSLP, which contributes to the pathogenesis of asthma [7]. The cigarette smoke [8] and other allergen exposures in the environment can trigger the reactive oxygen species (ROS) production [9] and can cause damage to cell components such as the mitochondrial damage [10], which leads to the programmed cell death such as apoptosis in BECs. The initiation of the aberrant apoptosis after injury may be one of the mechanisms that activate the airway inflammation in asthma [11–14]. The aberrant apoptosis in BECs can disrupt the integrity of the epithelial barriers [15] and can expose airways and lungs to excess pathogens or environmental allergens [16], which ultimately leads to the progression of asthma. A report indicated that the level of aberrant BECs apoptosis was related to the severity of asthma in asthma patients [17].

Apoptosis is obligatorily associated with the function of mitochondria [18–20]. Mitochondrial permeability transition pore (MPTP) is a multiprotein complex made up of cyclophilin-D (CyP-D), adenine nucleotide translocator (ANT) and voltage-dependent anion channel (VDAC), and it located between the inner and outer mitochondrial membranes [21]. The oxidative stress can promote the opening of MPTP, allowing small molecules below 1.5 KDa to permeate through the inner mitochondrial membrane, which leads to the loss of the mitochondrial membrane potentials (MMP), the uncoupling of the respiratory chain, the impaired synthesis of ATP, the swelling of the mitochondrial matrix, the rupture of the outer mitochondrial membrane, the release of pro-apoptotic factors from the intermembrane space, and to trigger an apoptotic cascade [22–23]. MPTP is the hub of the mitochondrial pathway of apoptosis, and its opening and closing is regulated by the Bcl-2 Protein Family and peripheral benzodiazepine receptors (PBRs) located in the outer mitochondrial membrane [24].

PBRs, also called translocator protein (TSPO), were discovered and identified accidentally when peripheral tissues from rats were used as the negative control for the central nervous system in a radioactive ligand binding assay in 1977 [25]. Some studies have reported that PBRs mediate neuroprotective effects [26]. Accumulating evidences suggest that PBRs are also highly expressed in the lungs, bronchus and adrenal glands [27–28], and they are primarily located in the outer mitochondrial membrane. After the ligands binding, PBRs are engaged in the regulation of MPTP, apoptosis and the steroid hormone synthesis [29]. Less is understood regarding the mechanism of the regulation of the mitochondrial function and apoptosis of BECs by PBR as well as its ligands in the treatment of asthma. In this study, we investigated the mechanism of PBR as well as its two classical synthetic ligands, agonist Ro5-4864 and antagonist PK 11,195, in the treatment of asthma in vitro and in vivo. We aimed to shed light on providing a new therapeutic target against asthma.

## Methods

### In vitro study

#### Cells

Cell line: human bronchial epithelioid cells [16HBE HBE135-E6E7, ATCC® CRL-2741 (authenticated by STR and tested for mycoplasma contamination), purchased on 05/06/2022 from Shanghai Fuheng Biotechnology Co., Ltd, China].

The cells were cultured in the cell culture vessels containing the Keratinocyte Medium (ScienCellTM, USA) and incubated in a cell culture incubator (37 °C, 5% CO2, saturated humidity).

### ROS detected by fluorescent probe DCFH-DA

The cells were divided into 8 groups: Control group, Lipopolysaccharide (LPS) group, LPS + Midazolam group, LPS + Ro5-4864 group, LPS + PK 11,195 group, LPS + PK 11,195 + Midazolam group, LPS + PK 11,195 + Ro5-4864 group and LPS + Cyclosporine-A group. 24 h prior to the addition of LPS (8 µg/ml, Biosharp, China), the cells were cultured with the Keratinocyte Medium containing Midazolam, Ro5-4864, PK 11,195 and Cyclosporine-A respectively in each group. Midazolam (Jiangsu Enhua, China), Ro5-4864 (MedChemExpress, USA), PK 11,195 (MedChemExpress, USA) and Cyclosporine-A (MedChemExpress, USA) were diluted in the Keratinocyte Medium containing 0.1% (v/v%) DMSO, achieving final concentrations of 2 µM, 10 µM, 10 µM, and 10 µM, respectively. After con-incubated with LPS for 24 h (Control group was received the equivalent volume of the vehicle solution), the culture medium was discarded. Subsequently, DCFH-DA working buffer (5 µM, UElandy, China) was added to each well and con-incubated at 37 °C for 30 min at dark. The images of the cells were captured under the identical exposure condition using a fluorescent microscope (100×, Olympus IX73), and the average fluorescence intensity of each group was then calculated and analyzed by the Image J software. The stronger fluorescence intensity indicated the higher level of the intracellular ROS.

### Cell apoptosis detected by flow cytometry

The cells were washed twice and centrifuged after detached by EDTA-free trypsin solution (Gibco, USA). 100 µl of Binding Buffer (1×) was added to resuspend the cell pellets. 5 µl of Annexin V-FITC and 10 µl of PI (AnnexinV-FITC/PI Apoptosis Detection Kit, Meilunbio, China) were added and con-incubated at 37 °C for 15 min at dark. The FITC channel (excitation wavelength 490 nm, emission wavelength 525 nm) and the PerCP channel (excitation wavelength 535 nm, emission wavelength 615 nm) were selected. A double-negative control group (unstained sample) was used to adjust the voltages of the Forward Scatter (FSC) and the Side Scatter (SSC), and two single-positive control groups (samples stained with FITC and PI separately) were used as compensation controls to compensate for the spectral overlap. A quadrant gate was set, and four regions were defined (Q1: Annexin V-/PI-, Q2: Annexin V+/PI-, Q3: Annexin V+/PI+, Q4: Annexin V-/PI+). The overall apoptosis rate (early and late stages) of each group was recorded.

### Co-localization of the cell nuclear, TSPO and mitochondria detected by immunofluorescence

Add Mito-Tracker Red CMXRos working buffer (200nM, Beyotime, China) to a 60 mm culture dish and incubate at 37 °C for 30 min at dark. The cells were washed three times and fixed with 4% paraformaldehyde at 37 °C for 30 min at dark. Add immunostaining permeabilization buffer (Triton X-100, Beyotime, China) and incubate at room temperature for 10 min at dark. Add immunostaining blocking buffer (QuickBlock™, Beyotime, China) and incubate at room temperature for 30 min at dark. Remove the blocking buffer, add TSPO first-antibody working solution (1:200, Abcam, UK) and incubate at 4 °C at dark overnight. Wash the cells, add second-antibody conjugated FITC working solution (1:200, Abbkine, USA) and incubate at room temperature for 60 min at dark. Add the mounting medium containing DAPI and capture images using a fluorescent microscope (100×).

### MMP (ΔΨ) detected by fluorescent probe Mito-Tracker Red CMXRos

The cells were divided into 4 groups: Control group, LPS group, LPS + Ro5-4864 group and LPS + PK 11,195 group. Mito-Tracker Red CMXRos working buffer (200nM, Beyotime, China) was added to each well and con-incubated at 37 °C for 30 min at dark. Capture images under the identical exposure condition using a fluorescent microscope (100×). The average fluorescence intensity of each group was then calculated and analyzed by the Image J software. The fluorescence intensity reflects ΔΨ, with the stronger fluorescence intensity indicating the higher level of ΔΨ.

### Mitochondrial swelling detected by the absorbance reading

The cells were trypsinized and counted to ensure equal cell numbers in each group in subsequent procedures. The mitochondria were extracted using a mitochondrial extraction kit (Solarbio, China). After resuspended with Lysis Buffer, the cells were subjected to 30–40 cycles of the grinding with the glass homogenizer on ice. The supernatant was collected after centrifugation at 1000 rcf for 5 min, and the cytoplasmic protein was obtained after the centrifugation of the supernatant at 12,000 rcf for 10 min. After removing the supernatant, the mitochondria were precipitated at the bottom. The mitochondrial precipitate was resuspended with Wash Buffer, and 100 µl of the mitochondrial suspension was added to a 96-well plate. 10 µl of CCK-8 solution was added to each well for the absorbance reading at 540 nm. The lower absorbance indicated the higher level of the mitochondrial swelling.

### Subcellular structures detected by transmission electron microscope (TEM)

The cells were collected and fixed in 2.5% glutaraldehyde and 1% osmic acid sequentially. The cell masses were dehydrated with gradient ethanol and 100% acetone sequentially and embedded in pure 812 embedding agent after permeated with acetone-812 embedding mixed agent. The resin blocks were sectioned at 50 nm by an ultramicrotome (RMC PT-PC), stained with 2% uranyl acetate and 2.6% lead citrate and then used for observation of the subcellular structure under a TEM (hitachi HT7800).

### Western blot

The total cellular protein was extracted using RIPA buffer (Beyotime, China) containing the protease inhibitor PMSF. Subsequently, the protein concentration of each group was determined by the BCA method (BCA Protein Assay Kit, NCMbio, China) to ensure the equivalent gel loading. After the protein loading, the SDS-PAGE gel electrophoresis was performed, which followed by transferring the protein molecule from the SDS-PAGE gel to a PVDF membrane. The PVDF membrane was blocked with 5% skimmed milk blocking buffer at room temperature for 1 h, incubated with first-antibody working solution (1:1000, CST Rabbit mAb, USA) at 4℃ overnight and subsequently incubated with second-antibody working solution (1:2000, CST Anti-rabbit IgG, HRP-linked Antibody, USA) at room temperature for 1 h. The PVDF membrane was immersed in a chemiluminescent substrate (ECL, Biosharp), and a greyscale image was obtained from the imaging system. The relative protein expression level was quantified as the ratio of the band density of the target protein to the reference protein, and it was expressed as the fold change.

### RT-qPCR

The total RNA of cells was extracted using the SteadyPure Universal RNA Extraction Kit (Agbio, China). For RNA extraction, the gDNA Eraser Mini Column and DNase I in the SteadyPure Universal RNA Extraction Kit (Agbio, China) were used to remove the genomic DNA. Before reverse transcription, the gDNA Clean Reaction Mix Ver.2 in the Evo M-MLV RT Mix Kit (Agbio, China) was used to remove the residual genomic DNA. 1 µg RNA per well was reversely transcribed into cDNA (20 µl per well) using the Evo M-MLV RT Mix Kit, and 2 µl per well of cDNA was used for RT-qPCR in each group using the SYBR Green Premix Pro Taq HS qPCR Kit III (Agbio, China) and PCR instrument (QuantStudio 5). The primer sequences were as follows: BCL-2-F: ATGTGTGTGGAGAGCGTCAA, BCL-2-R: CGGTTCAGGTACTCAGTCATCC, BAX-F: GGTTGTCGCCCTTTTCTACTTTG, BAX-R: GTCCAGCCCATGATGGTTCT, β-actin-F: TGGCACCCAGCACAATGAA, β-actin-R: CTAAGTCATAGTCCGCCTAGAAGCA. The relative gene expression level was quantified by 2^−ΔΔCt^ method and expressed as the fold change.

### In vivo study

#### Animals

The animal experiments were complied with the requirements of the Animal Experiment Ethics Committee and granted ethical approval by the Ethics Committee of Guangzhou University of Chinese Medicine (number: 20,221,026,003). Female C57/BL6J mice (6 to 8 week-old, 18–22 g, purchased from the Animal Experiment Center of Guangzhou University of Chinese Medicine, China) were housed at ambient temperature of 20–26℃, relative humidity of 40–70% and 12/12 hours light-dark schedule under a SPF barrier environment. All mice received autoclaved food and water without restrictions. Mice were all anesthetized by injecting intraperitoneally with 10% pentobarbital sodium before exsanguinated.

Mice were assigned to 5 groups randomly (*n* = 6): Control group, Ovalbumin (OVA) group, OVA + Ro5-4864 group, OVA + PK 11,195 group and OVA + Dexamethasone group. On day 1 and day 8, mice were sensitized by injecting intraperitoneally with 200 µl of sensitization solution containing 150 µg OVA (Sigma, USA) and 4 mg Imject Alum (Thermo Fisher Scientific, USA), and mice in Control group were injected with 200 µl of the PBS. From day 9 to 18, mice were injected intraperitoneally with the pretreatment drugs every 3 days: Ro5-4864 (3 mg/kg), PK 11,195 (3 mg/kg) and Dexamethasone (3 mg/kg), and mice in Control group and OVA group were received equivalent volume of vehicle (10%DMSO-PBS). From day 15, mice were challenged with PBS containing OVA on 6 consecutive days, alternating between the intranasal instillation (100 µg OVA/40µl PBS) and the nebulization (100 mg OVA/10 ml PBS) until sacrificed on day 20, and mice in Control group were received equivalent volume of PBS. Referring to some previous studies in which Ro5-4864 (3 mg/kg) and PK-11,195 (3 mg/kg) were administered via injecting intraperitoneally in a mouse disease model [30], we selected the dosage of 3 mg/kg for Ro5-4864 and PK-11,195 in the treatment of the mice in this study.

### ELISA

After anaesthetized, the mice were exsanguinated by the orbital veins, and the blood was coagulated and centrifuged to obtain the sera. The ELISA kit (Jiang Su MEIMIAN, China) was used, and blank wells, standard wells and sample wells were set separately. The standards and samples were added to the Microelisa stripplate and incubated at 37℃ for 30 min. Aspirate the liquid in wells, wash five times, add the HRP-conjugated detection antibody and incubate at 37℃ for 30 min. Add the substrate solution and incubate at 37℃ for 10 min at dark. Add the stop solution to stop the color reaction and read the absorbance of each well at 450 nm. The standard density and the OD value were used to generate the standard curve and the equation. Calculate the sample density with the OD value of the sample.

### Bronchoalveolar lavage

After the mice were sacrificed, the trachea was exposed. An indwelling needle cannula was inserted into the trachea and ligated for fixation. Bronchoalveolar lavage was conducted twice with 0.8 ml of PBS using a 1 ml-syringe. Bronchoalveolar lavage fluid (BALF) was collected and centrifuged, and the supernatant was obtained for the cytokines examination by ELISA. The cell sediments were resuspended with 100 µl of PBS, and 10 µl of the cell suspension was used for the cell counting. Additionally, 50 µl of the cell suspension was dropped into a cytocentrifuge (Cence TXD3), and the cell suspension was centrifuged at 3000 rpm for 15 min to ensure the even distribution of cells on the glass slide. Subsequently, the slide was air-dried, and the cells on the slide were fixed with methanol for 15 min. The slide was rinsed with PBS for 1 min, air-dried and stained with 1 ml of Wright-Giemsa staining solution (Solarbio, China) for 5 min. The microscopic examination (40×) was conducted using the Digital pathology scanning system (3D HISTEC, Pannoramic MIDI).

### Histopathological examination

The lung tissues of mice were fixed in 4% paraformaldehyde for 48 h and then dehydrated with gradient ethanol. The dehydrated lung tissues were cleared in xylene for 1 h and then embedded in paraffin. The tissue blocks were sectioned at 4 μm thickness and attached to the slides tightly. The slides were stained with hematoxylin-eosin and then used for the histopathology study. Images (40×) were obtained by the Digital pathology scanning system.

### Statistical analysis

Statistical analysis was performed using the IBM SPSS Statistics 20.0. For the quantitative data, data are presented as means with standard deviations when normally distributed, and differences between data were analyzed using one-way ANOVA when followed the homogeneity of variance or using Welch ANOVA when not follow the homogeneity of variance. LSD test is used for post hoc multiple comparisons. Data are presented as medians with interquartile ranges when not normally distributed, and differences between data were analyzed using Kruskal-Wallis H test and using Bonferroni test for post hoc multiple comparisons. For categorical data, data are presented as frequencies with percentages, and differences between data were analyzed using Chi-square test. *p* < 0.05 was considered to be statistically significant.

## Results

### Intracellular ROS

**Fig. 1:**
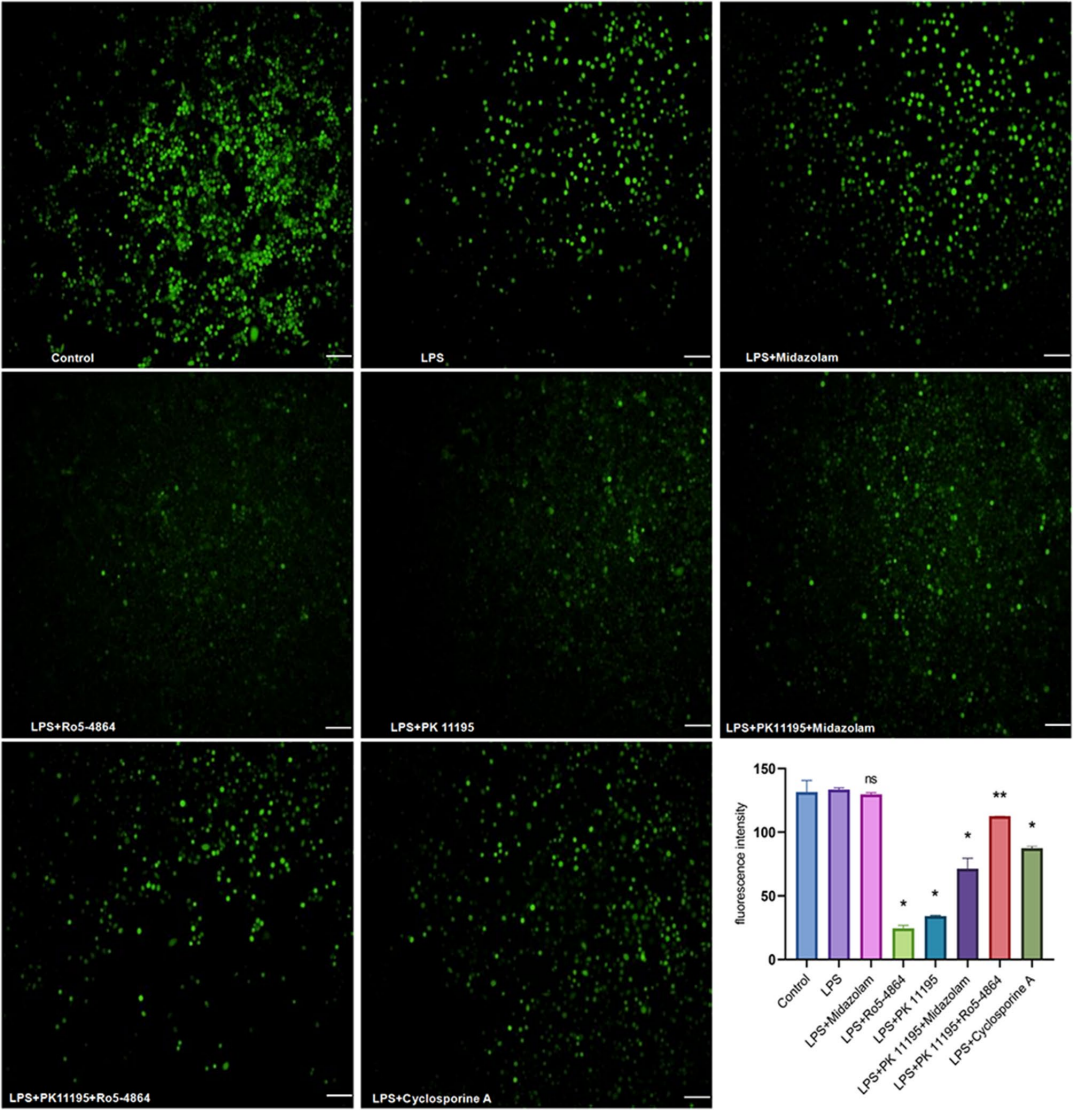
ROS detected by fluorescent probe DCFH-DA. The average fluorescence intensities were presented as mean ± SD. ^ns^*p*=0.421 vs. LPS group. ^*^p = 0.001 < 0.05 vs. LPS group. ^**^p = 0.002 < 0.05 vs. LPS group. Bar = 100 μm

The average fluorescence intensities: Control group (131.5450 ± 9.1995), LPS group (133.5750 ± 1.3647), LPS + Midazolam group (129.7600 ± 1.3152), LPS + Ro5-4864 group (24.5900 ± 2.2345), LPS + PK 11,195 group (34.1950 ± 0.4313), LPS + PK 11,195 + Midazolam group (71.4200 ± 8.0893), LPS + PK 11,195 + Ro5-4864 group (112.6250 ± 0.3748) and LPS + Cyclosporine-A group (87.4000 ± 1.7112). There was a significant difference in the average fluorescence intensities between the groups (*p* = 0.001). Compared with LPS group, LPS + Ro5-4864, LPS + PK 11,195, LPS + PK 11,195 + Midazolam, LPS + PK 11,195 + Ro5-4864 and LPS + Cyclosporine-A groups showed a decrease in average fluorescence intensities (*p* = 0.001, *p* = 0.001, *p* = 0.001, *p* = 0.002, *p* = 0.001). However, the average fluorescence intensity in LPS + Midazolam group was indistinguishable from LPS group (*p* = 0.421). (Fig. 1)

The PBR agonist Ro5-4864, antagonist PK 11,195 and Cyclosporine-A counteracted the production of the intracellular ROS induced by LPS, resulting in a decrease in the intracellular ROS level. However, the central benzodiazepine receptor (CBR) agonist Midazolam was unable to blunt the production of the intracellular ROS induced by LPS. Interestingly, Ro5-4864 and PK 11,195 exhibited the comparable counteracting effects on the production of the intracellular ROS induced by LPS, and their combined use did not show a synergistic effect compared with their alone use. Moreover, the antagonist PK 11,195 did not reverse the effect of the agonist Ro5-4864.

### Apoptosis rates of cells

**Fig. 2:**
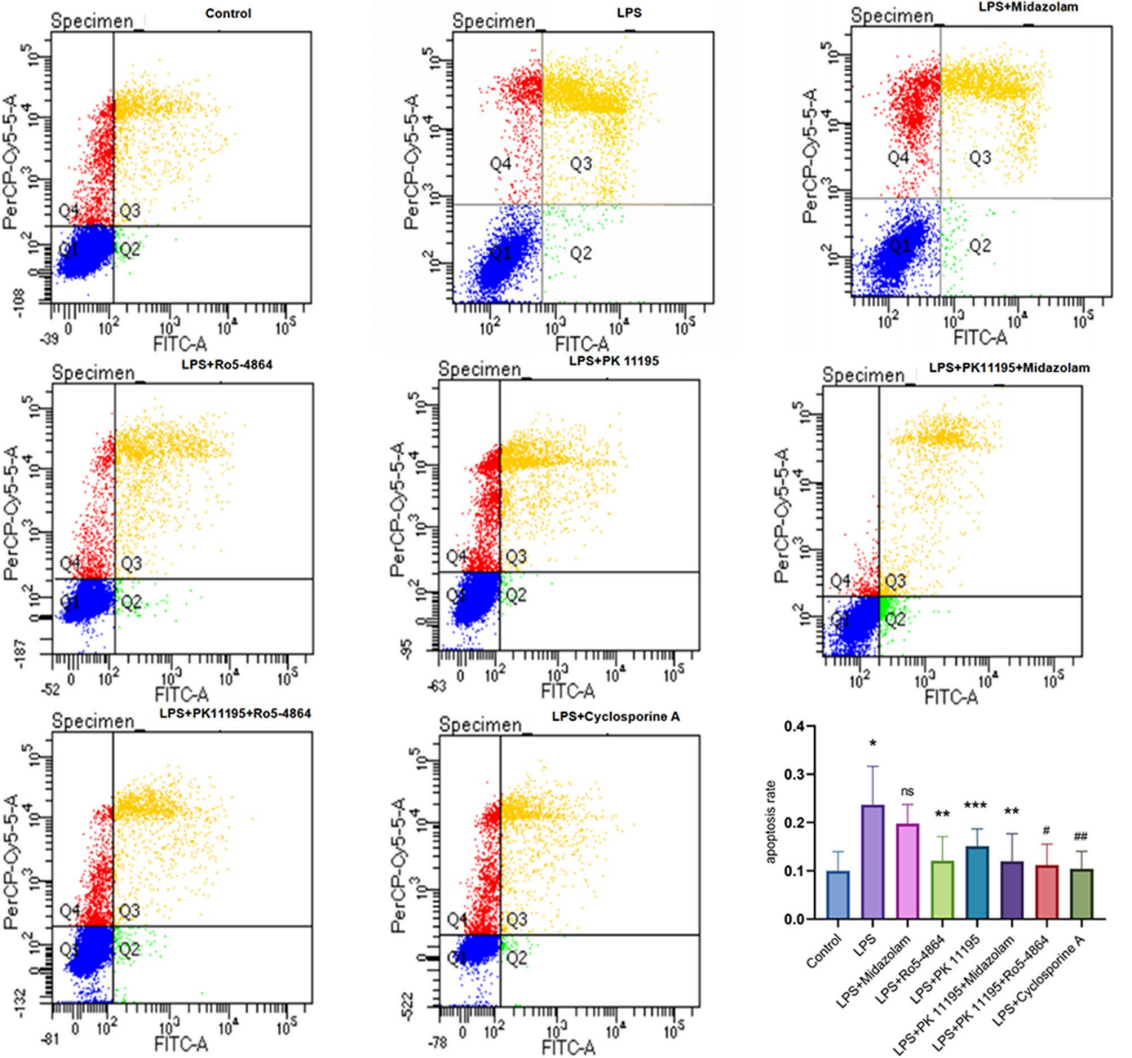
Cell apoptosis detected by flow cytometry. PerCP-Cy5_−_5: a kind of mixed dye. FITC: fluorescein isothiocyanate. The average total apoptosis rates were presented as mean ± SD. ^*^p = 0.004 < 0.05 vs. Control group. ^ns^*p*=0.349 vs. LPS group. ^**^p = 0.011 < 0.05 vs. LPS group. ^***^p = 0.05 vs. LPS group. ^#^*p* = 0.007 < 0.05 vs. LPS group. ^##^*p* = 0.005 < 0.05 vs. LPS group

The average total apoptosis rates: Control group (0.1000 ± 0.0397), LPS group (0.2370 ± 0.0796), LPS + Midazolam group (0.1980 ± 0.0397), LPS + Ro5-4864 group (0.1213 ± 0.0493), LPS + PK 11,195 group (0.1513 ± 0.0355), LPS + PK 11,195 + Midazolam group (0.1200 ± 0.0569), LPS + PK 11,195 + Ro5-4864 group (0.1120 ± 0.0435) and LPS + Cyclosporine-A group (0.1043 ± 0.0362). There was a significant difference in the average total apoptosis rates between the groups (*p* = 0.032). Compared with Control group, the average total apoptosis rate was increased in LPS group (*p* = 0.004). Compared with LPS group, LPS + Ro5-4864, LPS + PK 11,195, LPS + PK 11,195 + Midazolam, LPS + PK 11,195 + Ro5-4864 and LPS + Cyclosporine-A groups showed a decrease in the average total apoptosis rates (*p* = 0.011, *p* = 0.05, *p* = 0.011, *p* = 0.007, *p* = 0.005). There was no statistically significant difference in the average total apoptosis rates between LPS + Midazolam group and LPS group (*p* = 0.349). (Fig. 2)

The PBR agonist Ro5-4864, antagonist PK 11,195 and Cyclosporine-A exhibited an inhibitory effect on LPS-induced cell apoptosis, leading to a decrease in the average total apoptosis rates. However, the CBR agonist Midazolam did not show an anti-apoptotic effect. Similar to the results of the intracellular ROS detection, Ro5-4864 and PK 11,195 exhibited the comparable anti-apoptotic effects, and their combined use did not show a synergistic effect. Moreover, the antagonist PK 11,195 did not abrogate the anti-apoptotic effect of the agonist Ro5-4864 on cells treated with LPS.

### Co-localization of the cell nuclear, TSPO and mitochondria

**Fig. 3:**
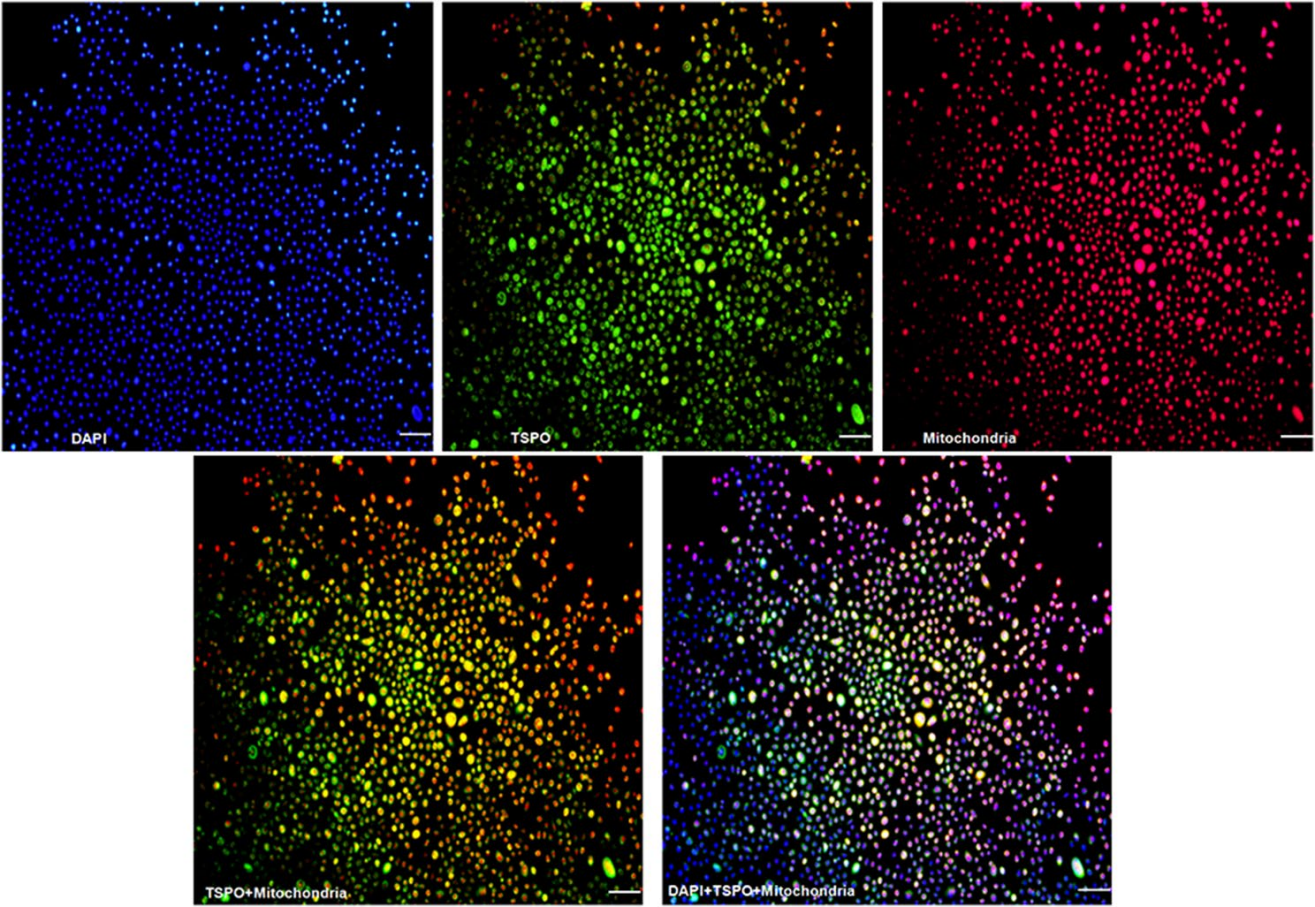
Co-localization of the cell nuclear, TSPO and mitochondria detected by immunofluorescence. Blue: the cell nuclear stained with DAPI, Green: the TSPO labeled with FITC, Red: the cell mitochondria stained with Mito-Tracker Red CMXRos, Yellow: the superposition of the green fluorescence (TSPO) and the red fluorescence (mitochondria), Purple: the superposition of the blue fluorescence (nuclear) and the red fluorescence (mitochondria). Bar = 100 μm

Under the fluorescence microscopy, the cell nuclear stained with DAPI emitted blue fluorescence. The TSPO labeled with FITC emitted green fluorescence, and the cell mitochondria stained with Mito-Tracker Red CMXRos emitted red fluorescence. The superposition of images of the green fluorescence (TSPO) and the red fluorescence (mitochondria) displayed yellow fluorescence, which indicated the co-localization of the TSPO and mitochondria. The TSPO was predominantly distributed on the cell mitochondria. (Fig. 3)

#### MMP (ΔΨ) of cells

**Fig. 4:**
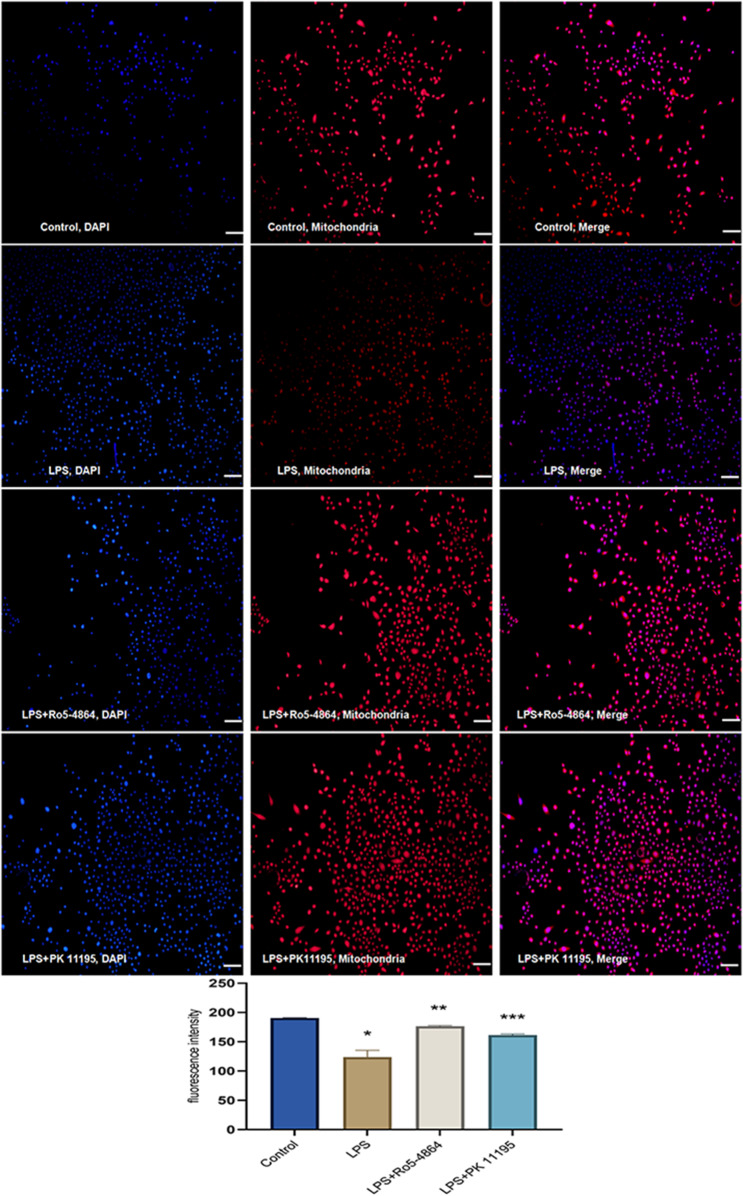
MMP (ΔΨ) detected by fluorescent probe Mito-Tracker Red CMXRos. The average fluorescence intensities were presented as mean ± SD. ^*^p = 0.001 < 0.05 vs. Control group. ^**^p = 0.001 < 0.05 vs. LPS group. ^***^p = 0.003 < 0.05 vs. LPS group. Bar = 100 μm

The average fluorescence intensity values: Control group (190.5200 ± 0.3818), LPS group (123.6800 ± 11.6531), LPS + Ro5-4864 group (176.5800 ± 1.1314) and LPS + PK 11,195 group (161.2800 ± 2.0223). The average fluorescence intensities exhibited statistically significant differences between the groups (*p* = 0.001). Compared with Control group, the average fluorescence intensity decreased in LPS group (*p* = 0.001). In contrast, LPS + Ro5-4864 and LPS + PK 11,195 groups showed an increase in average fluorescence intensities compared with LPS group (*p* = 0.001, *p* = 0.003). (Fig. 4)

The PBR agonist Ro5-4864 and the antagonist PK 11,195 alleviated the decrease of ΔΨ induced by LPS. The effects of Ro5-4864 and PK 11,195 on counteracting the decrease of ΔΨ were comparable.

### The absorbance of the cellular mitochondria

**Fig. 5:**
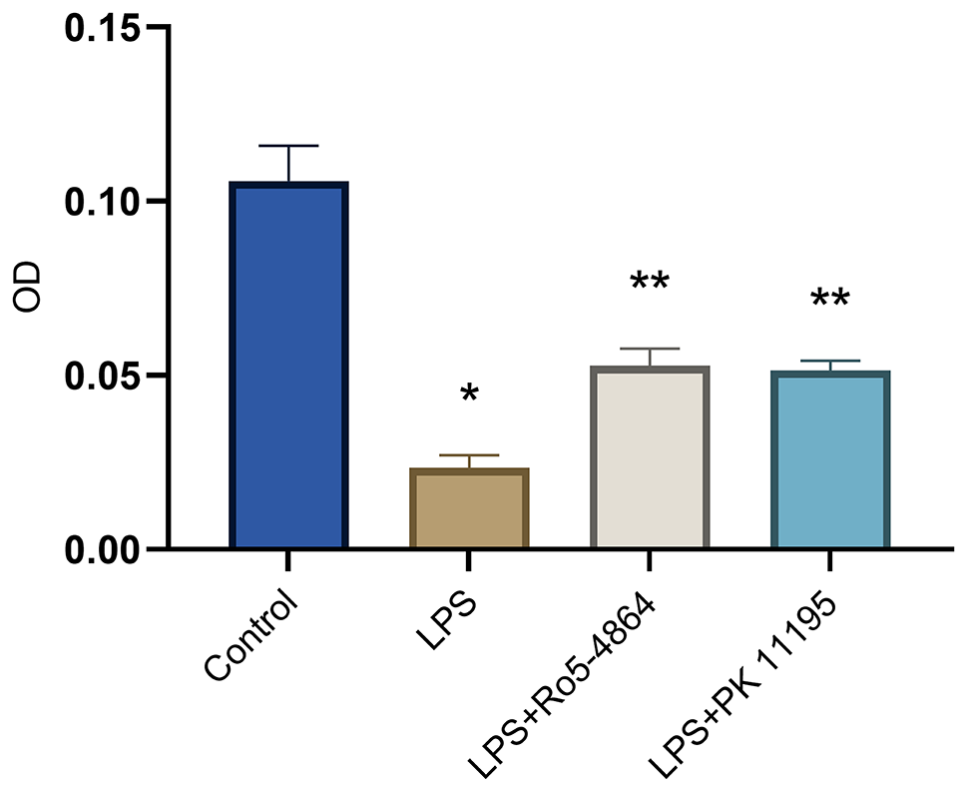
Mitochondrial swelling detected by absorbance. The OD values were presented as mean ± SD. ^*^p = 0.001 < 0.05 vs. Control group. ^**^p = 0.001 < 0.05 vs. LPS group

The OD values: Control group (0.1058 ± 0.0101), LPS group (0.0235 ± 0.0036), LPS + Ro5-4864 group (0.0528 ± 0.0048) and LPS + PK 11,195 group (0.0515 ± 0.0026). The differences of the OD values between the groups were statistically significant (*p* = 0.001). Compared with Control group, LPS group showed a decrease in the mitochondrial OD value (*p* = 0.001). In comparison with LPS group, LPS + Ro5-4864 group and LPS + PK 11,195 group exhibited an increase in the mitochondrial OD values (*p* = 0.001, *p* = 0.001). (Fig. 5)

The PBR agonist Ro5-4864 and the antagonist PK 11,195 both demonstrated a prophylactic effect on LPS-induced mitochondrial swelling, attenuating the extent of the cellular mitochondrial swelling. The agonist Ro5-4864 and the antagonist PK 11,195 achieved the same effect on counteracting the mitochondrial swelling.

### Subcellular structures of cells

**Fig. 6:**
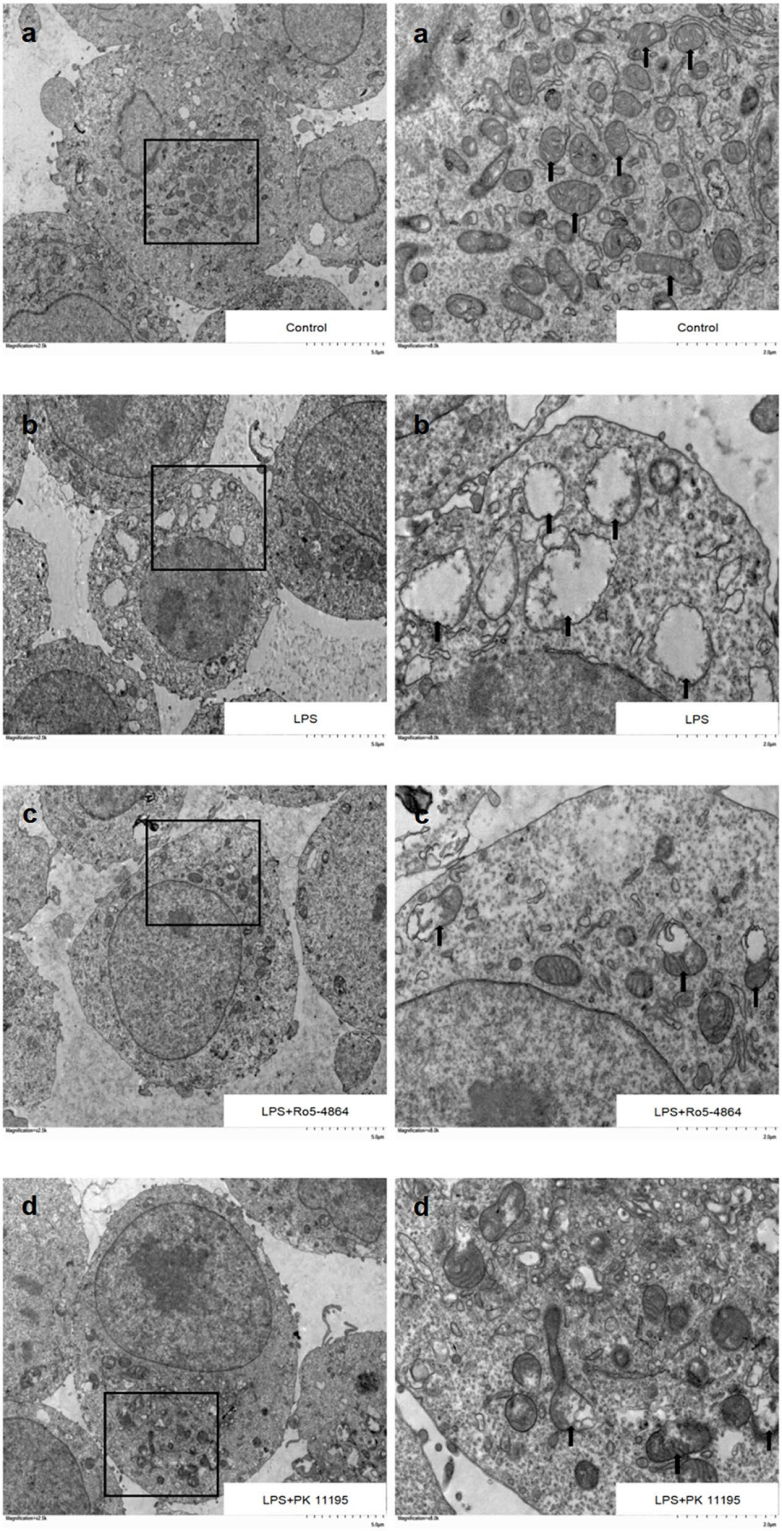
Subcellular structures detected by TEM. **a** There existed a large number of mitochondria with the normal structures and the intact cristae in Control group. **b** Mitochondrial injury was severe in LPS group. **c** The PBR agonist Ro5-4864 alleviated LPS-induced mitochondrial injury. **d** The PBR antagonist PK 11,195 alleviated LPS-induced mitochondrial injury

Control group: The double-layer structures of the nuclear membranes were clearly visible, and the chromatin was distributed evenly. A large number of the structurally intact mitochondria were existed with the shape of the long shuttle, and the mitochondrial cristae were intact, as shown by the arrows (Fig. 6a). LPS group: The cell volumes were smaller. There were a few blebbings on the cell membranes, and the number of organelles in the cytoplasm was reduced. The nuclear membrane was ruptured, and the chromatin was aggregated in the periphery of the nuclear membrane. The number of mitochondria was reduced, and the structures of the double membranes and the cristae were disappeared. The matrix of the mitochondria was swollen and vacuolated. The mitochondria suffered from severe injury, as shown by the arrows (Fig. 6b). LPS + Ro5-4864 group: The structures of the nucleus membranes were clear. The number of mitochondria was reduced, and some of the structures of the mitochondrial double membranes and the cristae were broken. A small part of the mitochondrial matrix was swollen and vacuolated. The injury of the cell ultrastructure in this group lessened compared with LPS group, as shown by the arrows (Fig. 6c). LPS + PK 11,195 group: The structures of the nucleus membranes were clear. The number of mitochondria was reduced. A small number of mitochondria with inner and outer membranes ruptured, and the matrix spillage led to a lesser extent of the injury compared with LPS group, as shown by the arrows (Fig. 6d).

### Protein expression levels

#### TSPO, VDAC and ANT

**Fig. 7:**
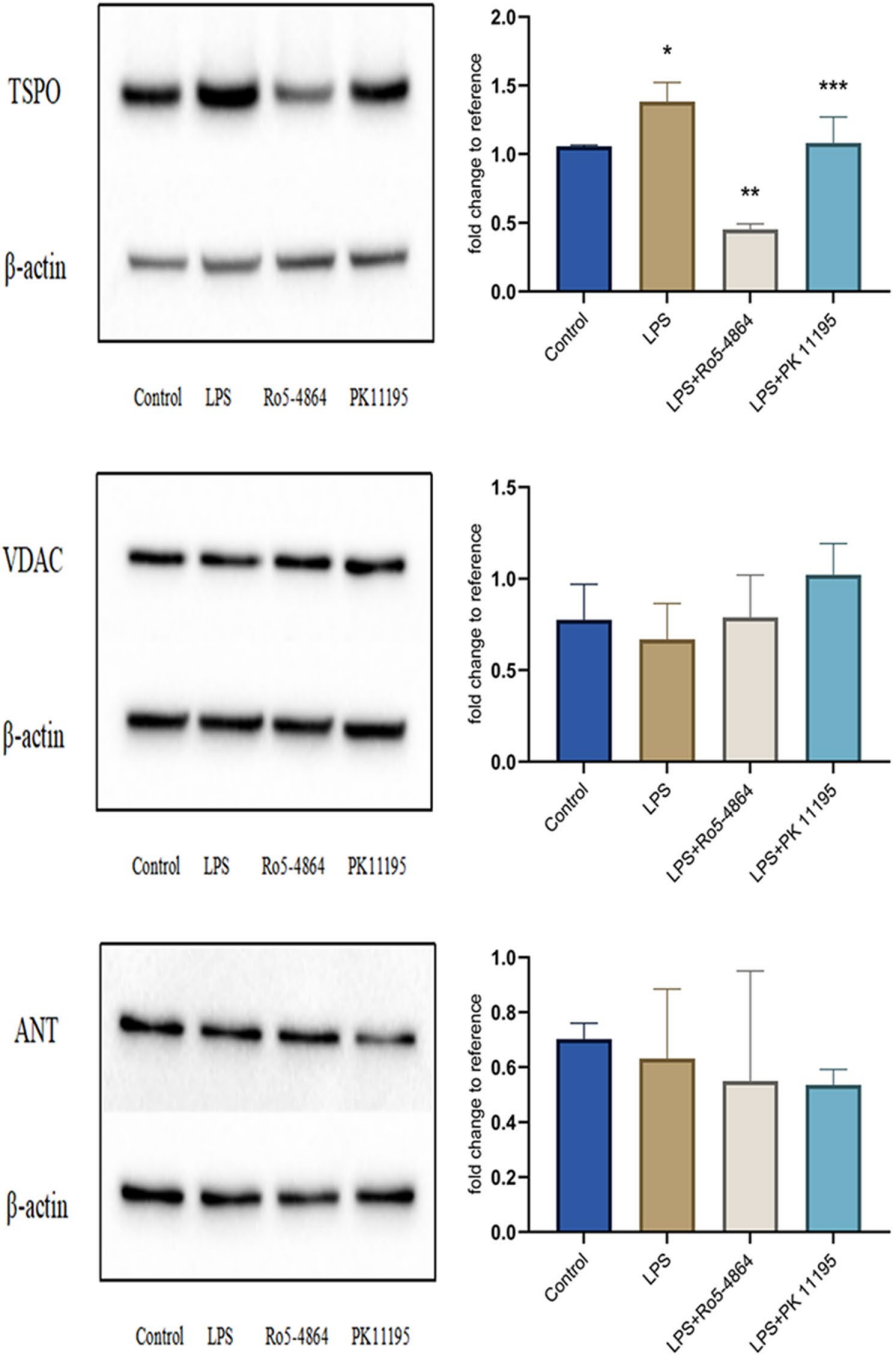
Western blot - TSPO (18KDa), VDAC (31KDa) and ANT (30KDa). The relative expression levels of proteins are presented as mean ± SD. TSPO: ^*^p = 0.01 < 0.05 vs. Control group. ^**^p = 0.001 < 0.05 vs. LPS group. ^***^p = 0.015 < 0.05 vs. LPS group. (The original uncropped blots were presented in the Additional file 1.)

The relative expression levels: TSPO: Control group (1.0567 ± 0.0091), LPS group (1.3824 ± 0.1406), LPS + Ro5-4864 group (0.4522 ± 0.0407) and LPS + PK 11,195 group (1.0828 ± 0.1886). VDAC: Control group (0.7762 ± 0.1928), LPS group (0.6682 ± 0.1966), LPS + Ro5-4864 group (0.7878 ± 0.2315) and LPS + PK 11,195 group (1.0212 ± 0.1706). ANT: Control group (0.7031 ± 0.0571), LPS group (0.6317 ± 0.2533), LPS + Ro5-4864 group (0.5495 ± 0.4010) and LPS + PK 11,195 group (0.5356 ± 0.0564). The differences of the TSPO expression between the groups were statistically significant (*p* = 0.001). The differences of the VDAC and ANT expression between the groups were not statistically significant (*p* = 0.249, *p* = 0.138). The expression level of TSPO increased in LPS group compared with Control group (*p* = 0.01) and decreased in LPS + Ro5-4864 group and LPS + PK 11,195 group compared with LPS group (*p* = 0.001, *p* = 0.015). However, the expression levels of VDAC and ANT did not differ between the groups. (Fig. 7)

LPS induced an increase in intracellular TSPO expression level. Pretreatments of cells with PBR agonist Ro5-4864 and antagonist PK 11,195 were all made a decrease in TSPO expression level, and the expression levels of VDAC and ANT were not affected.

#### Ratio of BCL-2 to BAX

**Fig. 8:**
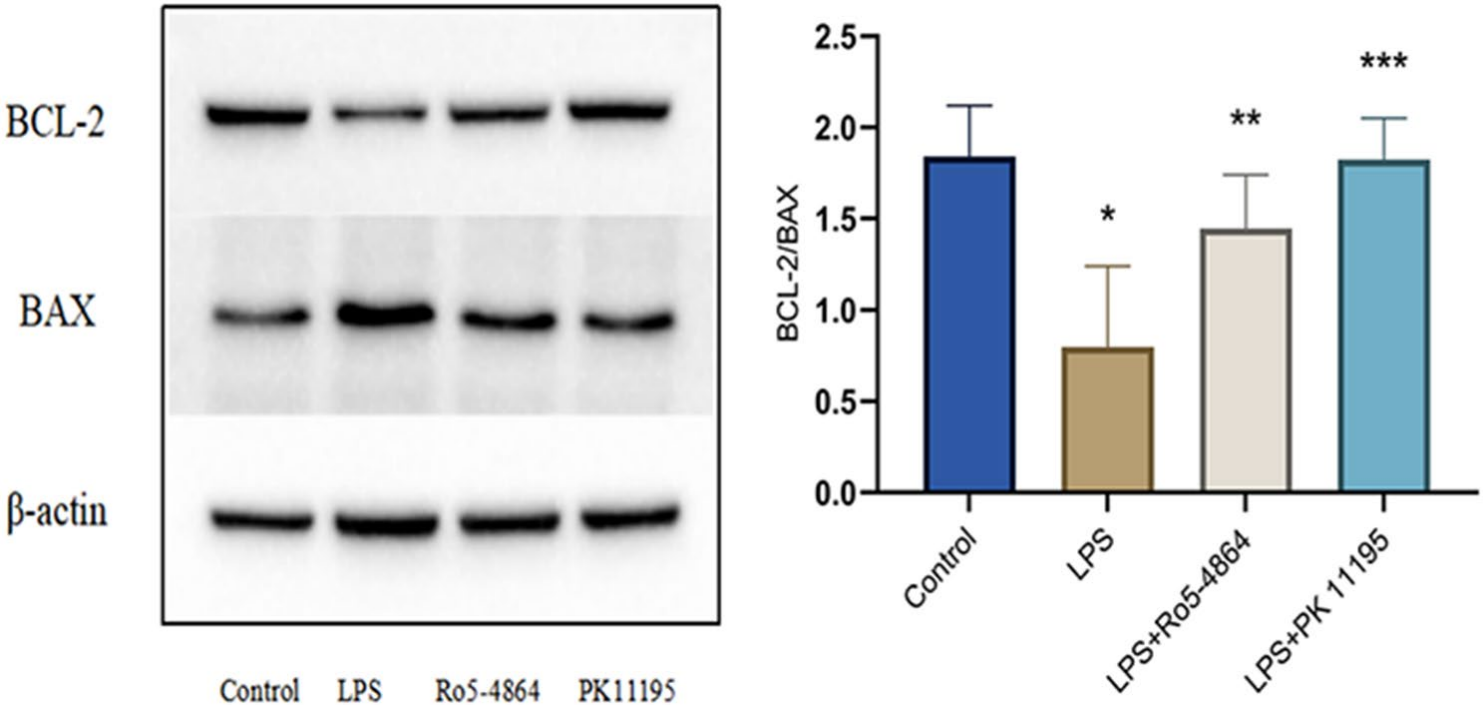
Western blot - Ratio of BCL-2 (52KDa) to BAX (21KDa). The relative expression levels of proteins are presented as mean ± SD. ^*^p = 0.004 < 0.05 vs. Control group. ^**^p = 0.038 < 0.05 vs. LPS group. ^***^p = 0.004 < 0.05 vs. LPS group. (The original uncropped blots were presented in the Additional file 1.)

The ratios of BCL-2 to BAX: Control group (1.8401 ± 0.2798), LPS group (0.7975 ± 0.4439), LPS + Ro5-4864 group (1.4461 ± 0.2938) and LPS + PK 11,195 group (1.8249 ± 0.2255). The differences of the ratios of BCL-2 to BAX between the groups were statistically significant (*p* = 0.013). The ratio of BCL-2 to BAX decreased in LPS group compared with Control group (*p* = 0.004) and increased in LPS + Ro5-4864 group and LPS + PK 11,195 group compared with LPS group (*p* = 0.038, *p* = 0.004). (Fig. 8)

LPS could lead to a decrease in the ratio of BCL-2 to BAX, which indicated that the pro-apoptotic protein was dominant. PBR agonist Ro5-4864 and antagonist PK 11,195 were found to reverse the ratio of BCL-2 to BAX, forming the predominance of expression of the anti-apoptotic protein.

#### Cytochrome C

**Fig. 9:**
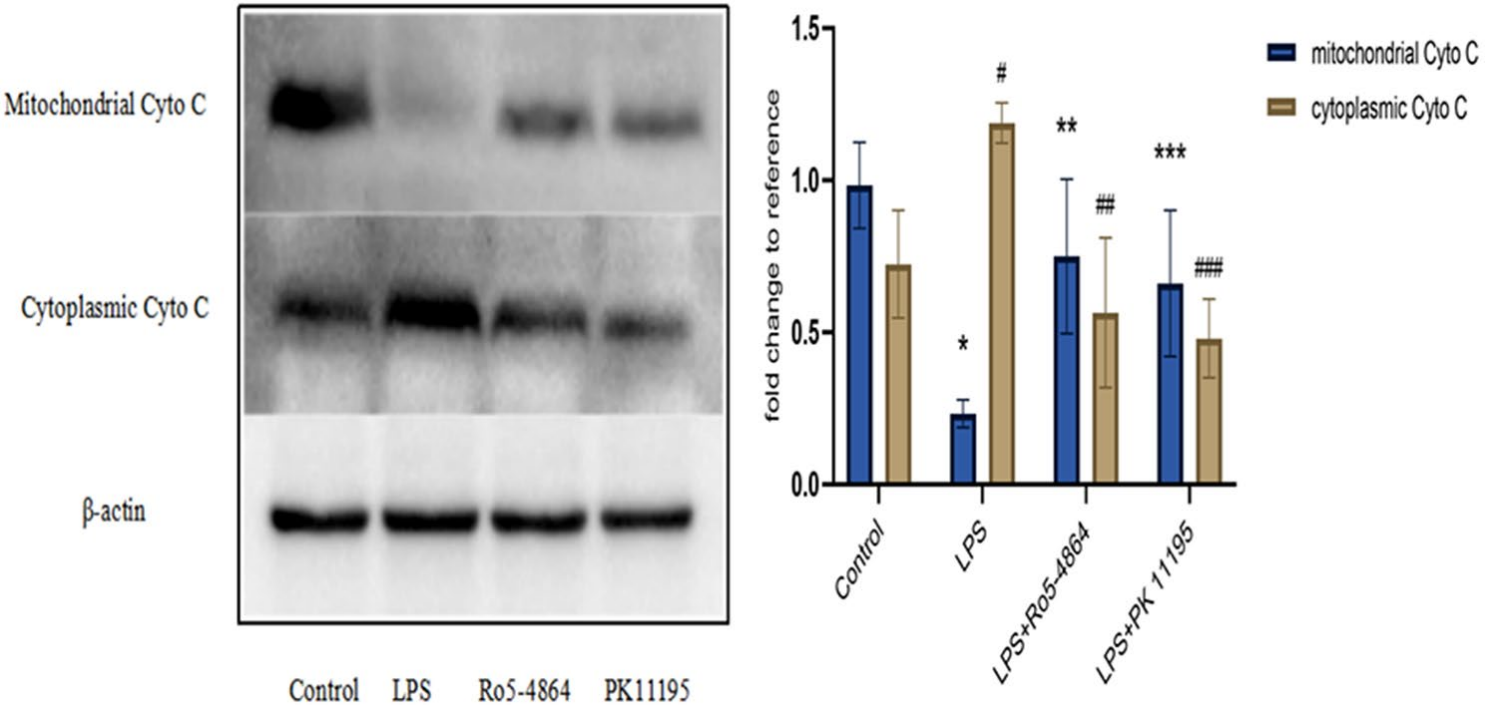
Western blot - Cytochrome C (15KDa). The relative expression levels of proteins are presented as mean ± SD. In the mitochondria: ^*^p = 0.001 < 0.05 vs. Control group. ^**^p = 0.01 < 0.05 vs. LPS group. ^***^p = 0.025 < 0.05 vs. LPS group. In the cytoplasm: ^#^*p* = 0.01 < 0.05 vs. Control group. ^##^*p* = 0.002 < 0.05 vs. LPS group. ^###^*p* = 0.001 < 0.05 vs. LPS group. (The original uncropped blots were presented in the Additional file 1.)

The relative expression levels: In the mitochondria: Control group (0.9829 ± 0.1415), LPS group (0.2324 ± 0.0455), LPS + Ro5-4864 group (0.7493 ± 0.2537) and LPS + PK 11,195 group (0.6606 ± 0.2399). In the cytoplasm: Control group (0.7237 ± 0.1772), LPS group (1.1884 ± 0.0666), LPS + Ro5-4864 group (0.5644 ± 0.2465) and LPS + PK 11,195 group (0.4798 ± 0.1294). The differences of the Cytochrome C expression in the mitochondria and cytoplasm between the groups were statistically significant (*p* = 0.008, *p* = 0.004). The expression level of the mitochondrial Cytochrome C decreased in LPS group (*p* = 0.001), and the cytoplasmic Cytochrome C increased in LPS group (*p* = 0.01) compared with Control group. The mitochondrial Cytochrome C increased in LPS + Ro5-4864 group and LPS + PK 11,195 group (*p* = 0.01, *p* = 0.025), and the cytoplasmic Cytochrome C decreased in LPS + Ro5-4864 group and LPS + PK 11,195 group (*p* = 0.002, *p* = 0.001) compared with LPS group. (Fig. 9)

LPS induced a decrease expression level of Cytochrome C in the mitochondria and an increase of Cytochrome C transferred into the cytoplasm. PBR agonist Ro5-4864 and antagonist PK 11,195 could reverse this situation, which lead to an increase of Cytochrome C in the mitochondria but a decrease in the cytoplasm.

#### Cleaved Caspase-7

**Fig. 10:**
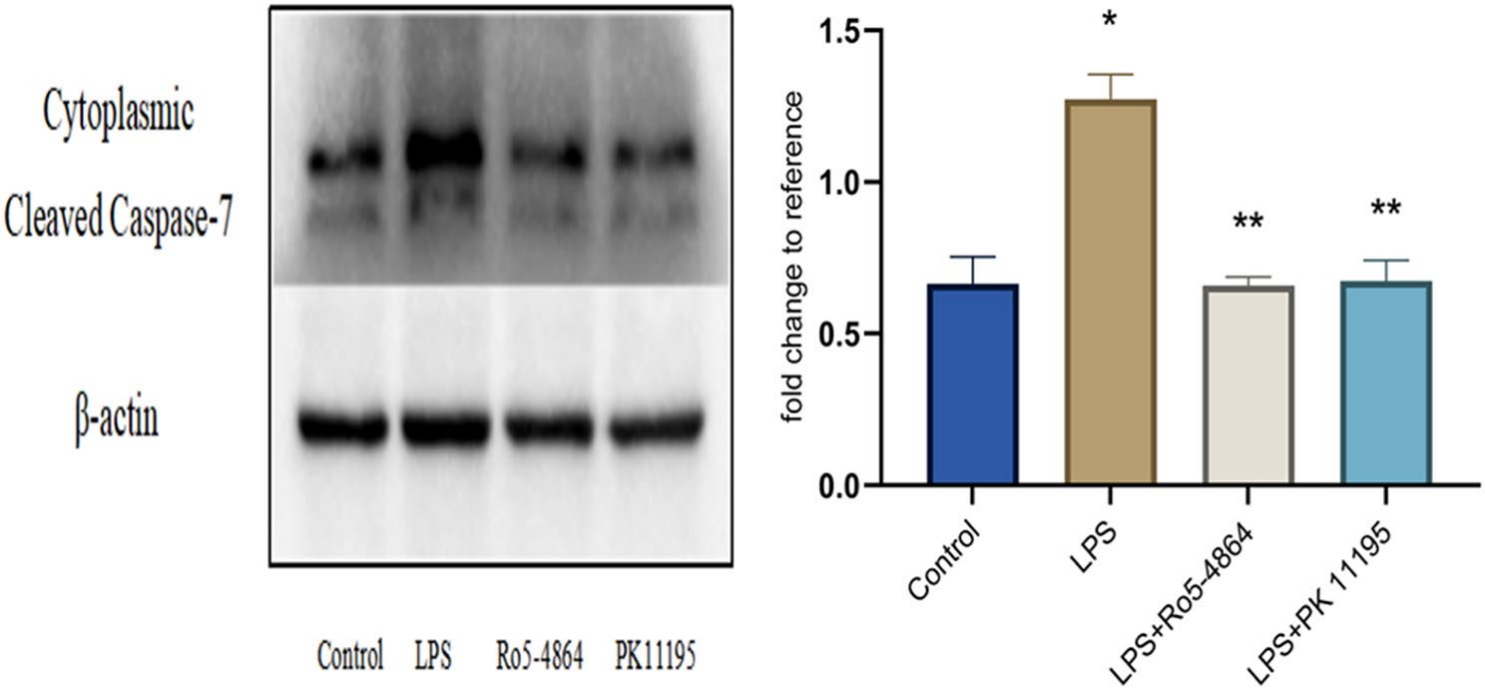
Western blot - Cleaved Caspase-7 (20KDa). The relative expression levels of proteins are presented as mean ± SD. ^*^p = 0.001 < 0.05 vs. Control group. ^**^p = 0.001 < 0.05 vs. LPS group. (The original uncropped blots were presented in the Additional file 1.)

The relative expression levels: Control group (0.6638 ± 0.0894), LPS group (1.2731 ± 0.0815), LPS + Ro5-4864 group (0.6586 ± 0.0287) and LPS + PK 11,195 group (0.6729 ± 0.0683). The differences of the cleaved Caspase-7 expression in the cytoplasm between the groups were statistically significant (*p* = 0.001). The expression level of cytoplasmic cleaved Caspase-7 increased in LPS group compared with Control group (*p* = 0.001) and decreased in LPS + Ro5-4864 group and LPS + PK 11,195 group compared with LPS group (*p* = 0.001, *p* = 0.001). (Fig. 10)

LPS induced an increase expression level of cleaved Caspase-7 in the cytoplasm. PBR agonist Ro5-4864 and antagonist PK 11,195 resulted in a decrease of cleaved Caspase-7 in the cytoplasm.

#### Gene expression levels

**Fig. 11:**
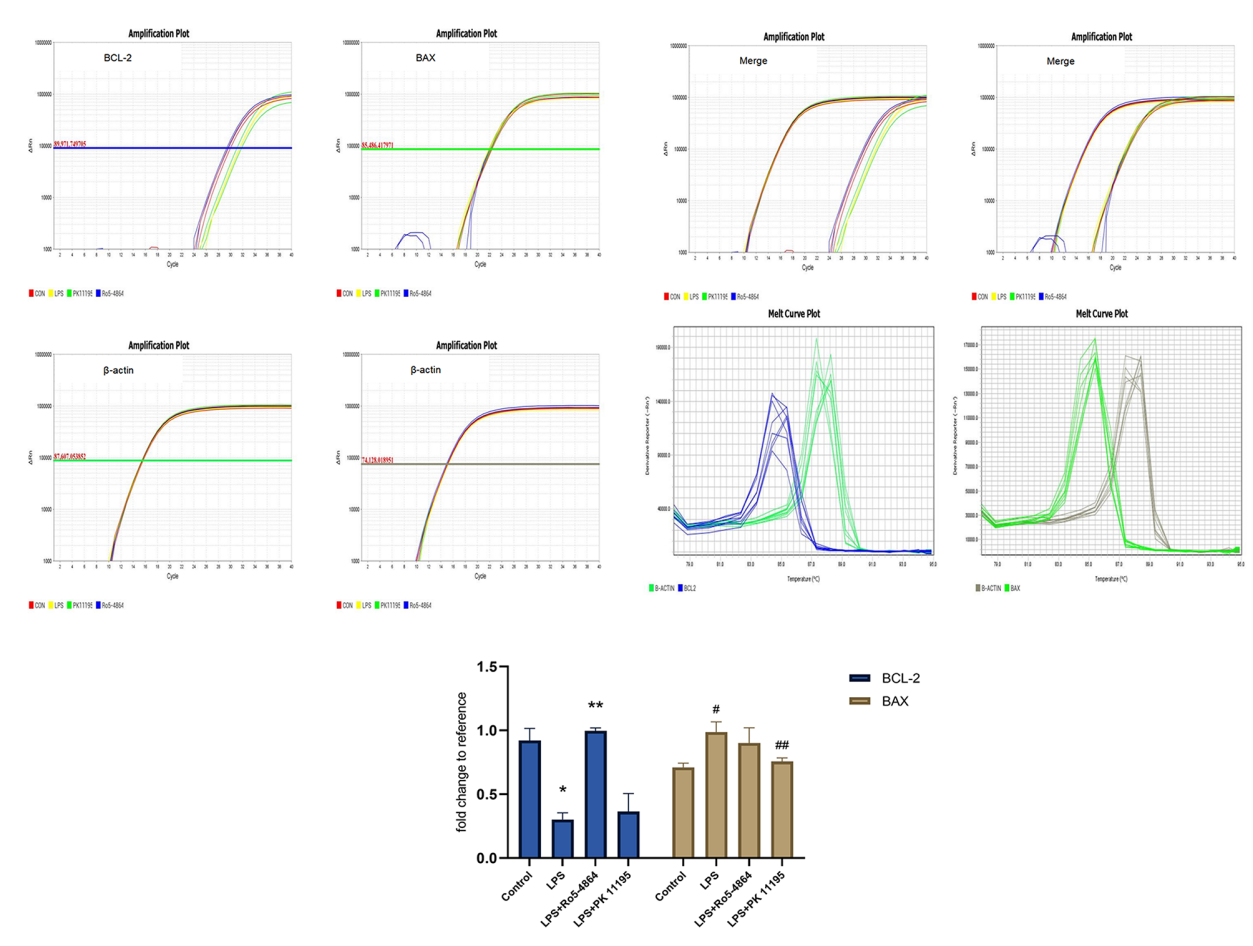
RT-qPCR - BCL-2 and BAX. The relative expression levels of genes are presented as mean ± SD. BCL-2: ^*^p = 0.001 < 0.05 vs. Control group. ^**^p = 0.001 < 0.05 vs. LPS group. BAX: ^#^*p* = 0.001 < 0.05 vs. Control group. ^##^*p* = 0.001 < 0.05 vs. LPS group

The relative expression levels: BCL-2: Control group (0.9200 ± 0.0962), LPS group (0.3017 ± 0.0515), LPS + Ro5-4864 group (0.9969 ± 0.0222) and LPS + PK 11,195 group (0.3636 ± 0.1416). BAX: Control group (0.7097 ± 0.0328), LPS group (0.9866 ± 0.0803), LPS + Ro5-4864 group (0.9009 ± 0.1190) and LPS + PK 11,195 group (0.7570 ± 0.0268). The differences of BCL-2 and BAX expression between the groups were statistically significant (*p* = 0.001, *p* = 0.005). The expression level of BCL-2 decreased in LPS group (*p* = 0.001), and BAX increased in LPS group (*p* = 0.001) compared with Control group. The expression level of BCL-2 increased in LPS + Ro5-4864 group (*p* = 0.001) compared with LPS group. The expression level of BAX decreased in LPS + PK 11,195 group (*p* = 0.001) compared with LPS group. (Fig. 11)

LPS induced a decrease in BCL-2 gene expression level and an increase in Bax gene expression level. PBR agonist Ro5-4864 led to an increase in BCL-2 gene expression level, and PBR antagonist PK 11,195 had no effect on the expression of BCL-2 gene. PBR antagonist PK 11,195 led to a decrease in Bax gene expression level, and PBR agonist Ro5-4864 had no effect on the expression of Bax gene.

### Serum IgE

**Fig. 12:**
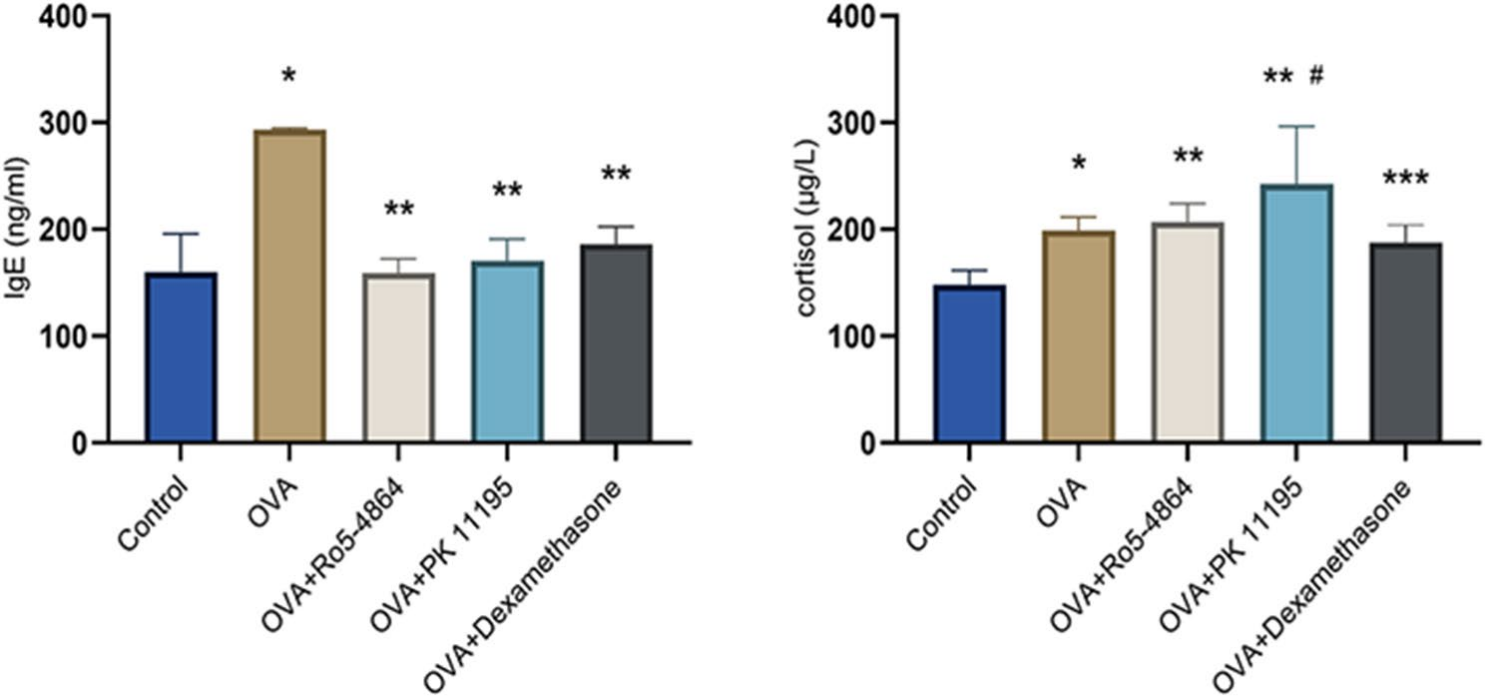
Serum ELISA. The concentration of IgE (ng/ml) and cortisol (µg/L) are presented as mean ± SD. IgE: ^*^p = 0.001 < 0.05 vs. Control group. ^**^p = 0.001 < 0.05 vs. OVA group. Cortisol: ^*^p = 0.004 < 0.05 vs. Control group. ^**^p = 0.001 < 0.05 vs. Control group. ^***^p = 0.021 < 0.05 vs. Control group. ^#^*p* = 0.011 < 0.05 vs. OVA group

The concentration of IgE (ng/ml): Control group (160.1333 ± 35.7417), OVA group (293.4667 ± 1.3663), OVA + Ro5-4864 group (159.3000 ± 13.0346), OVA + PK 11,195 group (170.6333 ± 20.1536) and OVA + Dexamethasone group (186.3000 ± 16.0468). The differences of the concentration of IgE were statistically significant (*p* = 0.001). The concentration of IgE increased in OVA group compared with Control group (*p* = 0.001) and decreased in OVA + Ro5-4864 group, OVA + PK 11,195 group and OVA + Dexamethasone group compared with OVA group (*p* = 0.001, *p* = 0.001, *p* = 0.001). ( Fig. 12)

The concentration of IgE increased in OVA group and decreased after the treatment of the glucocorticoid. Both of Ro5-4864 and PK 11,195 made a decrease in the concentration of IgE in OVA-treated mice.

### Serum cortisol

The concentration of cortisol (µg/L): Control group (148.1855 ± 13.2973), OVA group (198.7231 ± 12.8816), OVA + Ro5-4864 group (206.9220 ± 17.0949), OVA + PK 11,195 group (242.4059 ± 53.9319) and OVA + Dexamethasone group (187.6344 ± 16.8501). The differences of the concentration of cortisol were statistically significant (*p* = 0.001). The concentration of cortisol increased in OVA group, OVA + Ro5-4864 group, OVA + PK 11,195 group and OVA + Dexamethasone group compared with Control group (*p* = 0.004, *p* = 0.001, *p* = 0.001, *p* = 0.021). Compared with OVA group, the concentration of cortisol increased in OVA + PK 11,195 group (*p* = 0.011). However, the differences among OVA group, OVA + Ro5-4864 group and OVA + Dexamethasone group were not statistically significant (*p* = 0.612, *p* = 0.494). (Fig. 12)

The concentration of cortisol increased in mice subjected to OVA (including OVA group, OVA + Ro5-4864 group, OVA + PK 11,195 group and OVA + Dexamethasone group) compared with Control group. PK 11,195 demonstrated much greater potency for the secretion of cortisol, and Ro5-4864 showed weak facilitation.

### IFN-γ, IL-5 and IL-17 in BALF

**Fig. 13:**
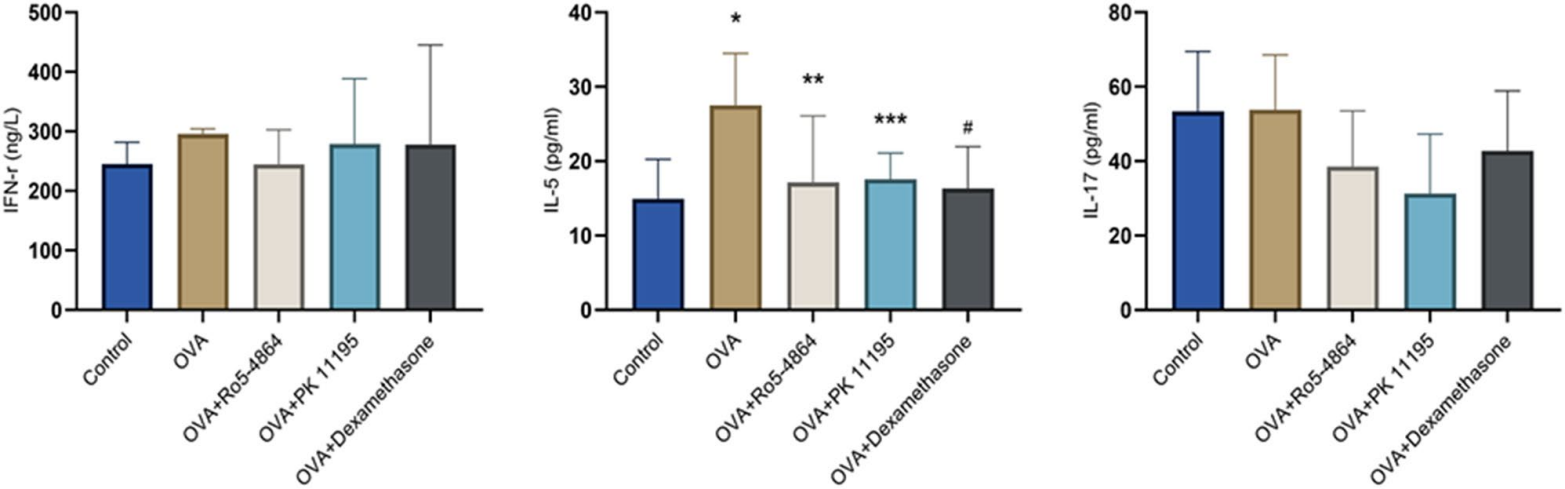
BALF ELISA. The concentration of IFN-γ (ng/L), IL-5 (pg/ml) and IL-17 (pg/ml) are presented as mean ± SD. IL-5: ^*^p = 0.002 < 0.05 vs. Control group. ^**^p = 0.009 < 0.05 vs. OVA group. ^***^p = 0.012 < 0.05 vs. OVA group. ^#^*p* = 0.005 < 0.05 vs. OVA group

The concentrations of the cytokines: IFN-γ (ng/L): Control group (245.1563 ± 36.7071), OVA group (295.6771 ± 8.5819), OVA + Ro5-4864 group (244.6354 ± 58.1822), OVA + PK 11,195 group (278.7500 ± 109.6336) and OVA + Dexamethasone group (277.9688 ± 167.3692). IL-5 (pg/ml): Control group (14.9401 ± 5.3042), OVA group (27.5118 ± 6.9649), OVA + Ro5-4864 group (17.1415 ± 8.9518), OVA + PK 11,195 group (17.5886 ± 3.4816) and OVA + Dexamethasone group (16.3192 ± 5.6276). IL-17 (pg/ml): Control group (53.3413 ± 16.1508), OVA group (53.8088 ± 14.7874), OVA + Ro5-4864 group (38.5978 ± 14.9246), OVA + PK 11,195 group (31.2927 ± 16.0279) and OVA + Dexamethasone group (42.7644 ± 16.1169). The differences of the concentration of IFN-γ and IL-17 between the groups were not statistically significant (*p* = 0.063, *p* = 0.086). The differences of the concentration of IL-5 between the groups were statistically significant (*p* = 0.015). The concentration of IL-5 increased in OVA group compared with Control group (*p* = 0.002) and decreased in OVA + Ro5-4864 group, OVA + PK 11,195 group and OVA + Dexamethasone group compared with OVA group (*p* = 0.009, *p* = 0.012, *p* = 0.005). **(**Fig. 13**)**

The concentration of IL-5 increased in OVA group and decreased after the treatment of the glucocorticoid. Both Ro5-4864 and PK 11,195 made a decrease in the concentration of IL-5 in OVA-treated mice, and the concentration of IFN-γ and IL-17 were not affected.

### Inflammatory cells in BALF

*Cell smears with Wright-Giemsa staining*

**Fig. 14:**
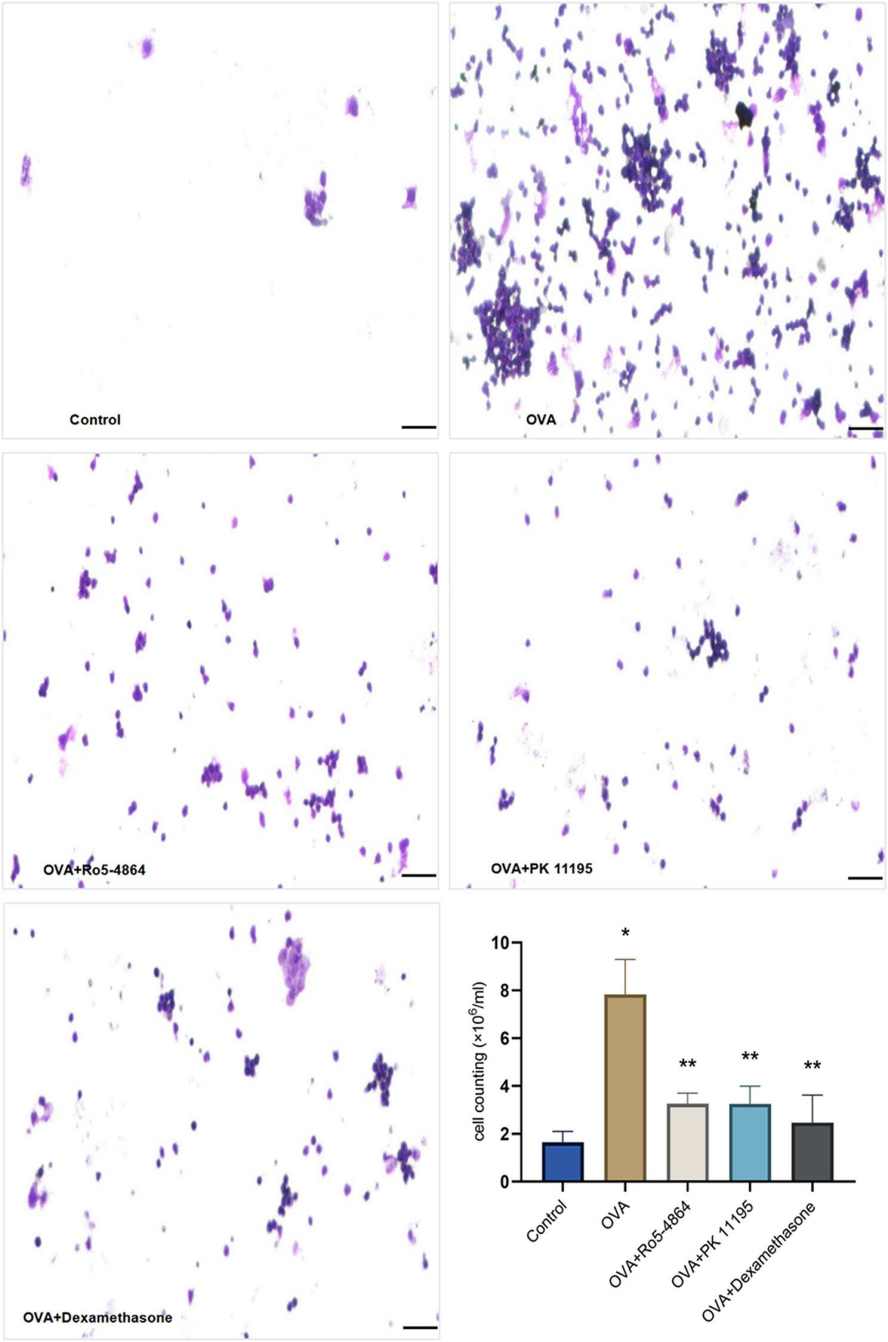
Inflammatory cells in BALF. Cell smears with Wright-Giemsa staining: The inflammatory cells were increased and aggregated on the BALF smear of OVA group, decreased and scattered after the treatment of the Dexamethasone. Both Ro5-4864 and PK 11,195 made a decrease and scatter of inflammatory cells on the BALF smear in OVA-treated mice. Cell counting: The total numbers of cells (10^6^/ml) in the BALF are presented as mean ± SD. ^*^p = 0.001 < 0.05 vs. Control group. ^**^p = 0.001 < 0.05 vs. OVA group. Bar = 50 μm

The erythrocytes mixed in BALF were lysed with RBC lysis buffer before BALF cells centrifugation, and only inflammatory cells (bluish-purple particles) were visible on the BALF smear. A few inflammatory cells were on the smear of Control group. The inflammatory cells were increased and aggregated on the smear of OVA group compared with Control group. Compared with OVA group, the inflammatory cells were decreased and scattered on the smear of OVA + Ro5-4864 group, OVA + PK 11,195 group and OVA + Dexamethasone group. (Fig. 14)

#### Cell counting

The total numbers of cells (10^6^/ml) in the BALF: Control group (1.6450 ± 0.4564), OVA group (7.8400 ± 1.4560), OVA + Ro5-4864 group (3.2600 ± 0.4445), OVA + PK 11,195 group (3.2517 ± 0.7447) and OVA + Dexamethasone group (2.4700 ± 1.1498). The differences of the total numbers of cells in the BALF were statistically significant (*p* = 0.001). The total numbers of cells increased in OVA group compared with Control group (*p* = 0.001) and decreased in OVA + Ro5-4864 group, OVA + PK 11,195 group and OVA + Dexamethasone group compared with OVA group (*p* = 0.001, *p* = 0.001, *p* = 0.001). (Fig. 14)

The total numbers of cells in the BALF increased in OVA group and decreased after the treatment of the glucocorticoid. Both Ro5-4864 and PK 11,195 made a decrease in the total numbers of cells in the BALF in OVA-treated mice.

### Histopathological analysis

**Fig. 15:**
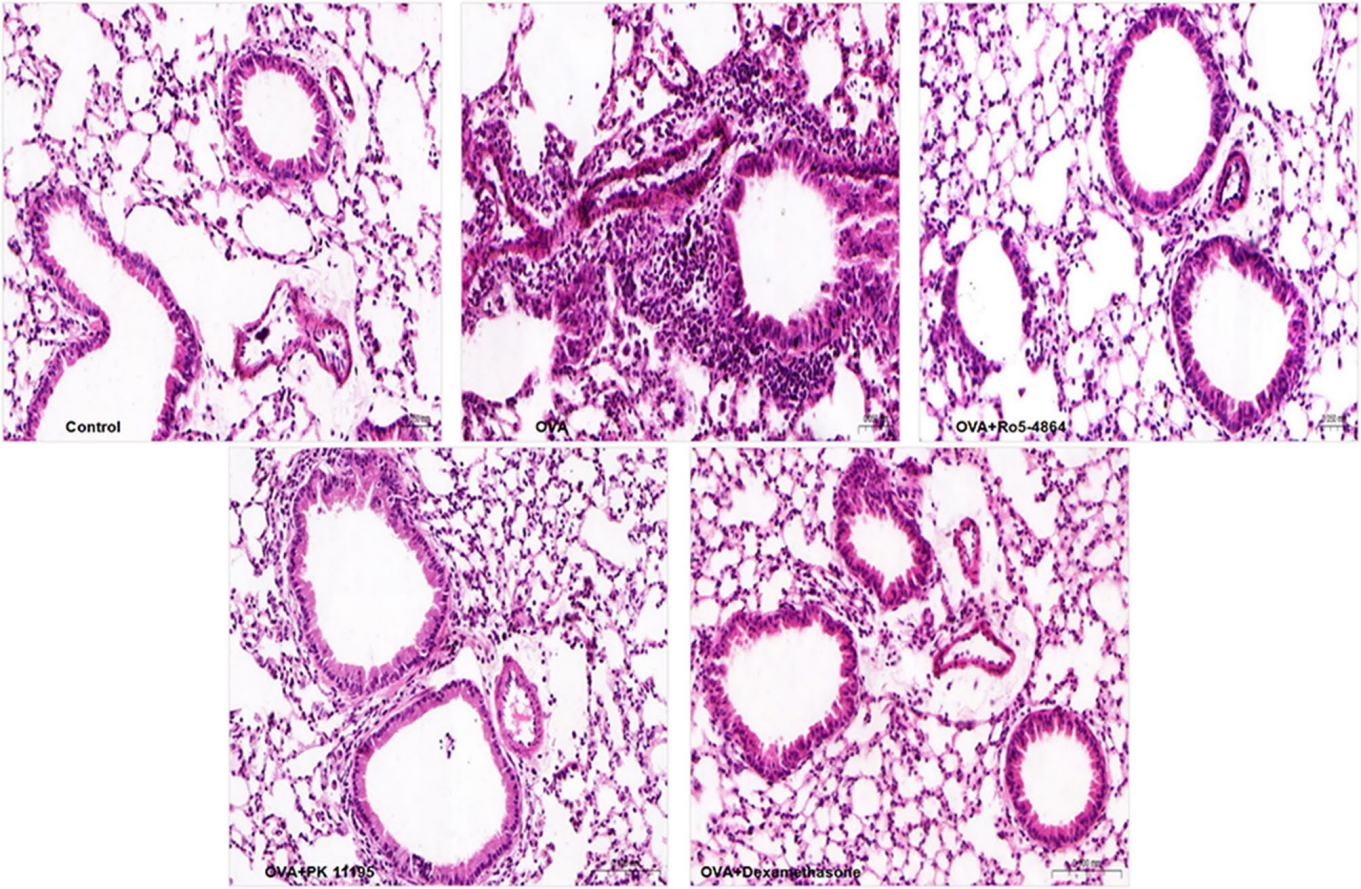
Histopathological examination. The lungs histopathological examination showed the injury of BECs and the infiltration of inflammatory cells in the peribronchial and submucosal tissue spaces in OVA group. The injury of BECs and the infiltration of inflammatory cells were abated after the treatments of Ro5-4864, PK 11,195 and Dexamethasone

In Control group, the BECs were arranged orderly, and the bronchial walls were thin. The structures of alveolar walls were complete, and few inflammatory cells could be seen in the peribronchial and submucosal tissue spaces. Compared with Control group, the structures of the BECs were disordered with the exfoliation of parts of the BECs. The submucosal tissue spaces were edematous, and the bronchial walls were thickened. The pronounced infiltration with inflammatory cells had extended to the peribronchial and submucosal tissue spaces in OVA group. Compared with OVA group, the basal lamina was intact with a lesser exfoliation of BECs and a diminution of inflammatory cells infiltration in the peribronchial and submucosal tissue spaces in OVA + Ro5-4864 group, OVA + PK 11,195 group and OVA + Dexamethasone group. (Fig. 15)

## Discussion

The cells were used to mimic the characteristics of the inflammatory injury of BECs on the condition of asthma in vitro. LPS has been widely used to induce the cell inflammation [31]. To investigate the mechanism of benzodiazepine receptor drugs against the inflammatory injury, we first detected the intracellular ROS and cell apoptosis. In order to distinguish CBR and PBR and to verify the specificity of ligands binding to receptors, we introduced the CBR agonist Midazolam [32], the PBR agonist Ro5-4864 and the PBR antagonist PK 11,195 [33]. We determined the optimum concentration and the time conditions for the remaining experiments after our pilot studies. Our results suggested that Ro5-4864, PK 11,195 and positive control Cyclosporine-A alleviated the production of ROS and apoptosis induced by LPS in BECs, which were in line with the results observed in the human retinal pigment epithelium [34]. Ro5-4864 and PK 11,195 showed a comparable effect separately, and the combination of the two did not show a stronger synergistic effect. The antagonist PK 11,195 did not reverse the effect of the agonist Ro5-4864. Our results differed mildly from those of another study which suggested that agonist Ro5-4864 had no protective effect on BECs [35], which may due to the different cell lines and the different pretreatment drugs and pretreatment time. Our results confirmed the plausible hypothesis that the binding sites and mechanisms of the two ligands were different but overlapped partially although both of them acted on PBRs [36]. Midazolam, a CBR agonist, did not decrease the level of intracellular ROS and the apoptosis rate, which indicated that CBR was structurally and pharmacologically distinct with PBR and that the anti-inflammatory effect was only related to PBRs. PBR agonist Ro5-4864 and antagonist PK 11,195 showed the same effect as Cyclosporine-A, a specific inhibitor of MPTP opening, on reducing ROS and anti-apoptosis, highlighting a role of MPTP opening as a response to the inflammation induced by LPS in BECs. PBRs may be the new therapeutic target against the inflammatory diseases such as asthma. It is postulated that PBR ligands play an anti-ROS and anti-apoptotic effect by inhibiting the opening of MPTP mediated by their receptors as the mechanism at least in part.

We verified the distribution of PBR in BECs, its subcellular localization in mitochondria and its elevated expression level in injured cells, which was consistent with previous reports [34]. Accordingly, we hypothesized that the anti-apoptotic effect mediated by PBR was related to the mitochondrial apoptotic pathway. We examined the relevant events of the mitochondrial apoptotic pathway. MMP (ΔΨ) is regulated by the MPTP [37]. When the MPTP is opening, water molecules entering the mitochondria can reduce the density of mitochondrial granules and can cause the mitochondrial swelling, which result in the varies of the mitochondrial absorbance reading. After the mitochondria extracted, the absorbance reading at a specific wavelength can reflect the degree of the mitochondrial swelling, and smaller absorbance values represent the more swollen mitochondria [23]. In addition to detecting the mitochondrial swelling indirectly, it is also possible to observe the subcellular structures such as mitochondria by TEM directly. Our results showed that both Ro5-4864 and PK 11,195 had mitochondrial protective effects, and another study also observed the mitochondrial protective effect of Ro5-4864 by TEM [38].

We demonstrated that LPS induced a decrease protein expression level in the ratio of BCL-2 to BAX, which meant the predominant pro-apoptotic protein promoted cell apoptosis after the inflammatory injury. Both Ro5-4864 and PK-11,195 reversed the ratio of anti-apoptotic to pro-apoptotic protein. We also confirmed that LPS induced a pronounced decrease protein expression level of Cytochrome C which is essential for the cell respiration in the mitochondria and an increase protein expression level of Cytochrome C in the cytoplasm, which elucidated the fact that LPS promoted Cytochrome C to transfer from the mitochondria to the cytoplasm, thus playing a role in the subsequent formation of apoptotic bodies, cleavage and activation of caspase-7, the ‘final apoptosis executor’ [39]. Moreover, Ro5-4864 and PK-11,195 could ameliorate the Cytochrome C transformation induced by LPS in the mitochondria and the cytoplasm and the caspase-7 activation induced by Cytochrome C. Our results suggested that Ro5-4864 and PK 11,195 possessed anti-apoptotic effects of the mitochondrial pathway.

To further solidify the mechanism of the anti-apoptotic effects of Ro5-4864 and PK 11,195, we examined the gene expression levels of BCL-2 and BAX. Our results suggested that LPS reduced the gene expression level of BCL-2 and increased the gene expression level of BAX, initiating cell apoptosis synergistically. PBR agonist Ro5-4864 reversed the BCL-2 gene, but not BAX gene, expression induced by LPS, and PBR antagonist PK 11,195 reversed the BAX gene, but not BCL-2 gene, expression induced by LPS. The cell survival is modulated by the balances between anti-apoptosis and pro-apoptosis, and is achieved by promoting the expression of anti-apoptotic factors and inhibiting the pro-apoptotic factors [40]. Our results of PCR further elucidated that Ro5-4864 and PK-11,195 exerted anti-apoptotic effects by regulating the gene expression levels of BCL-2 and BAX, respectively.

To establish the relevance of the above cell-based studies in vivo, glucocorticoid-sensitive asthma was set as the disease model for our animal experiment. Asthma is a chronic inflammatory disease which is generally considered to be driven by the type 2 inflammation mediated by the innate lymphoid cells-2 (ILC2) and the T helper cells-2 (Th2) [41]. The type 2 inflammation in asthma is initiated by ‘alarmin cytokines’ such as TSLP, IL-25, and IL-33 secreted by BECs when sensitized by allergens [7]. These cytokines act on their respective receptors IL-7Rα, IL-17RA/B and ST2 on ILC2 and Th2 cells, stimulating the differentiation of ILC2 and Th2 cells and the production of type 2 cytokines-IL-4, IL-5 and IL-13 [42]. IL-4 assists B cells to secret IgE. IL-5 promotes the activation and recruitment of eosinophils, and IL-13 elicits the mucus secretion [43]. Eosinophils release Major Basic Protein-1, Eosinophil Cationic Protein, Eosinophil Peroxidase and Eosinophil-derived Neurotoxin, which leads to the tissue injury around the airways [44]. For certain allergens, the ‘alarmin cytokines’ can also activate the non-type 2 inflammation pathways. Th17-related inflammation pathways dominated by Th17 cells and their products play a role in the activation and recruitment of neutrophils [45]. Additionally, the ‘alarmin cytokines’ can activate Th1-related inflammation pathways, which contributes to the accumulation of neutrophils and the airway inflammation [46]. These non-type 2 inflammation phenotypes could be the underlying nosogenesis of the severe asthma or refractory asthma [47]. Currently, about 50% of asthma cases are occupied by eosinophilic asthma associated with the type 2 inflammation, and this proportion exceeds 80% in pediatric patients [48]. The inflammation phenotype in glucocorticoid-sensitive asthma is primarily the type 2 inflammation pathways.

We detected the levels of IFN-γ, IL-5 and IL-17 in BALF, representing the Th1, Th2 and Th17-related inflammatory pathways and phenotypes respectively. Our results suggested that the concentration of IL-5 increased in the glucocorticoid-sensitive asthmatic mice and that the concentrations of IFN-γ and IL-17 maintained unchanged. The treatments of the glucocorticoid as well as Ro5-4864 and PK 11,195 were found to make a decrease in the concentration of IL-5, indicating an inhibitory effect on the type 2 inflammation in glucocorticoid-sensitive asthma.

Glucocorticoid is the conventional and effective drug in the treatment of asthma. We set dexamethasone as a positive control to verify the success of the asthma animal model and to compare the effectiveness of the PBR drugs used in the experiment with the classical positive control drug. Apart from the exogenous glucocorticoid, our body also synthesizes and secretes the endogenous cortisol. We detected the levels of the endogenous cortisol self-synthesized in mice, and the results suggested that the cortisol levels in sera of asthma models, including OVA group, OVA + Ro5-4864 group, OVA + PK 11,195 group and OVA + Dexamethasone group, increased compared with Control group. Above all, The OVA + Dexamethasone group treated with the glucocorticoid alone possessed the mildly increased level of the cortisol, which could be explained as the fact that mice sensitized and challenged by OVA could manifest a compensatory increase in the endogenous synthesis and secretion of the cortisol by the adrenal glands in order to prevent the progression of asthma, and the fact that the hypothalamic-pituitary-adrenal axis was subjected to feedback inhibition when the exogenous glucocorticoid was administered, which resulted in the suppression of the endogenous cortisol synthesis in the adrenal glands. Notably, after the treatment of PK 11,195, the serum cortisol increased significantly, far exceeding the compensatory increase, which indicated that PK 11,195 might play a role in promoting of the synthesis of the endogenous cortisol in the adrenal glands. In 2006, the HUGO Gene Nomenclature Committee designated the 18 kDa translocator protein as the new name for PBR due to its high density in the steroid hormone-secreting glands and its role in transporting the cholesterol to the mitochondria for the steroid hormones synthesis [49].

Currently, a number of studies have reported the relation between TSPO and various diseases. TSPO is involved in inhibiting the inflammatory responses in microglia [50]. Ro5-4864 can inhibit the formation of amyloid-like proteins in Alzheimer’s disease [51]. TSPO ligands exhibit protective effects on both cardiac and renal tissues injury induced by ischemia-reperfusion [52–53]. Our study is one of the few studies available that explore the relation between asthma and TSPO as well as its ligands. However, there are some limitations in our study. Firstly, there were no direct evidence of the mitochondrial channel opening or closing in vitro. Furthermore, the animal experiment focused solely on the asthma-related airway inflammation, without investigating the significant asthma features such as the mucus secretion and the airway remodeling. Finally, the results in vitro and in vivo could not be extrapolated to clinical patients directly.

## Conclusions

The findings of this study provided the evidence that TSPO could be a regulatory target for the mitochondrial function and apoptosis in BECs. Both the TSPO agonist Ro5-4864 and the antagonist PK 11,195 exerted the mitochondrial protective and anti-apoptotic effects and exerted a therapeutic effect on asthma mice.

### Electronic supplementary material

Below is the link to the electronic supplementary material.

Supplementary Material 1

## Data Availability

All data generated or analysed during this study are included in this published article.

## Abbreviations

ANT: adenine nucleotide translocator
AHR: airway hyperresponsiveness
BECs: bronchial epithelial cells.
BALF: bronchoalveolar lavage fluid.
CBR: central benzodiazepine receptor.
CyP-D: cyclophilin-D.
ILC2: innate lymphoid cells-2.
LPS: lipopolysaccharide.
MMP: mitochondrial membrane potentials.
MPTP: mitochondrial permeability transition pore.
OVA: ovalbumin.
PBRs: peripheral benzodiazepine receptors.
ROS: reactive oxygen species.
Th2: T helper cells-2.
TEM: transmission electron microscope.
TSPO: translocator protein.
VDAC: voltage-dependent anion channel.
