# Supplementary figures and images for: Mitochondria protective and anti-apoptotic effects of peripheral benzodiazepine receptor and its ligands on the treatment of asthma in vitro and vivo
Source: J Inflamm (Lond). 2024 Apr 19;21:11. doi: 10.1186/s12950-024-00383-0 (PMC11031857; doi:10.1186/s12950-024-00383-0)

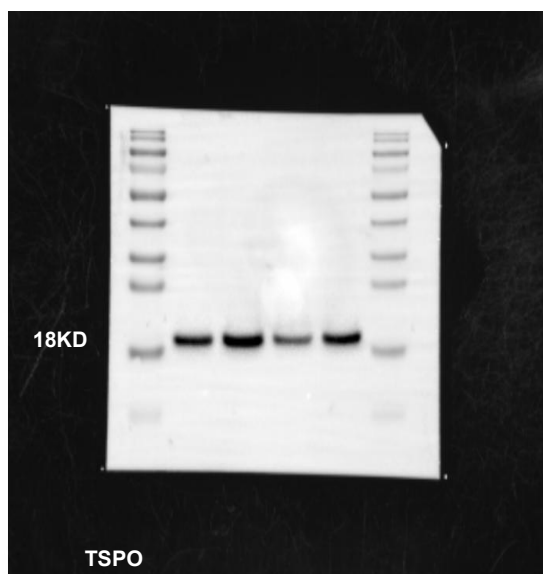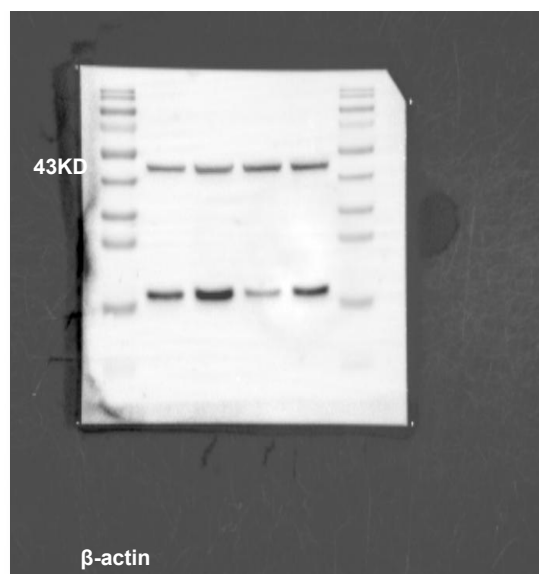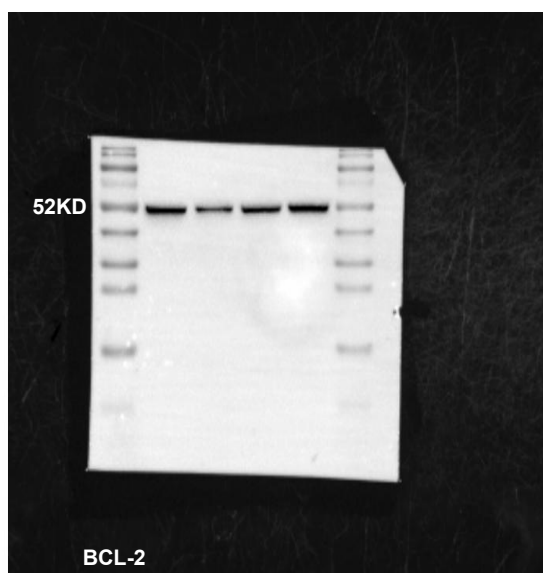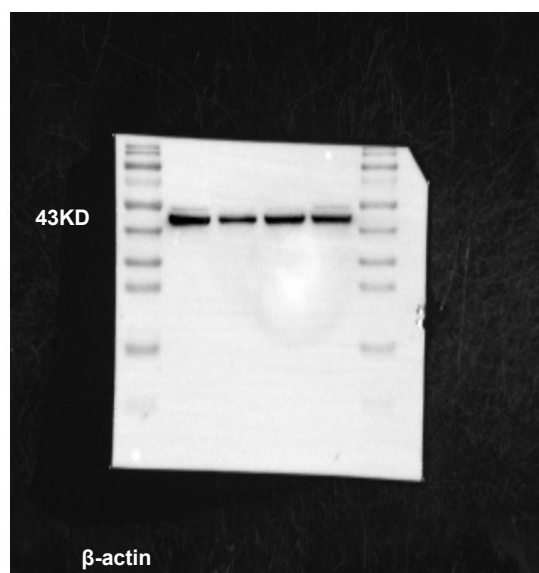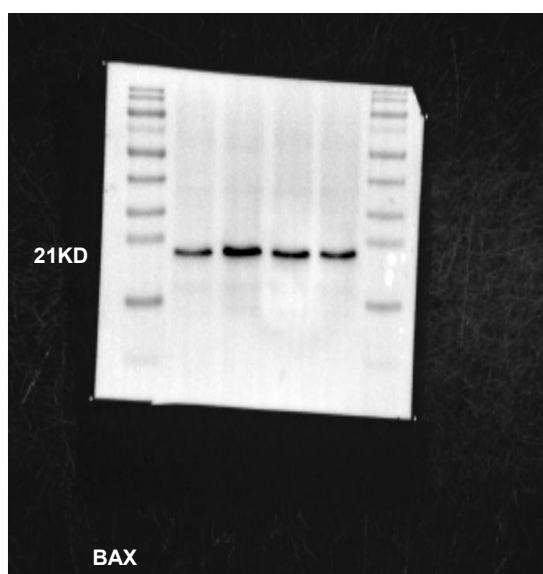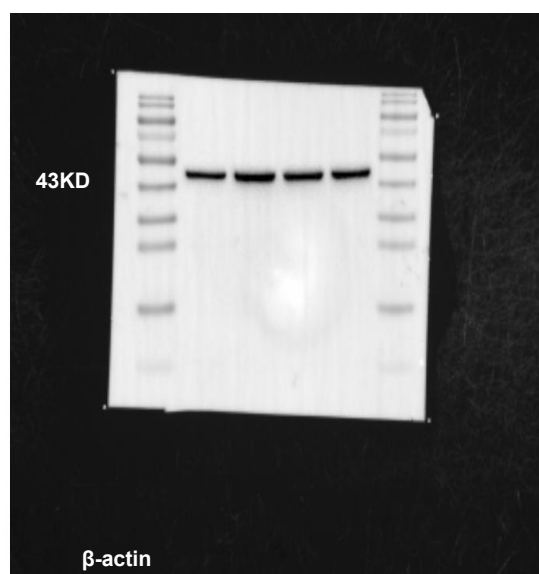

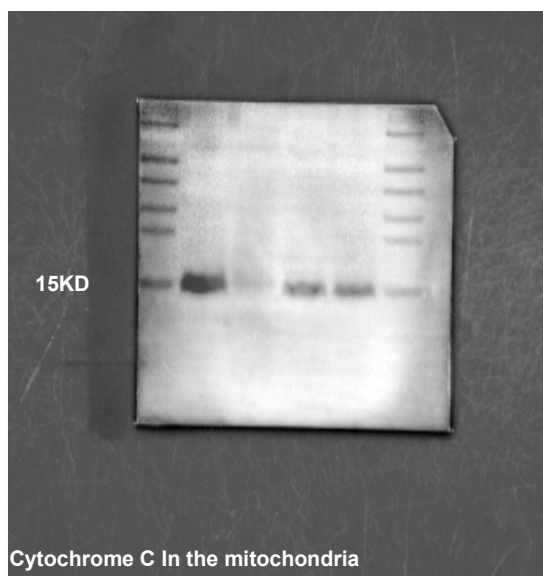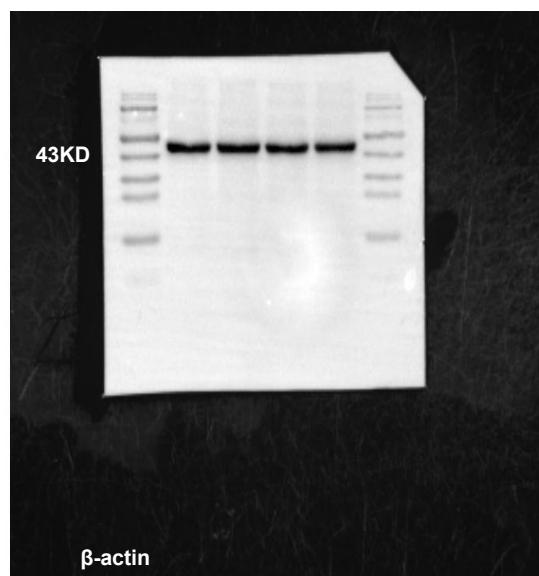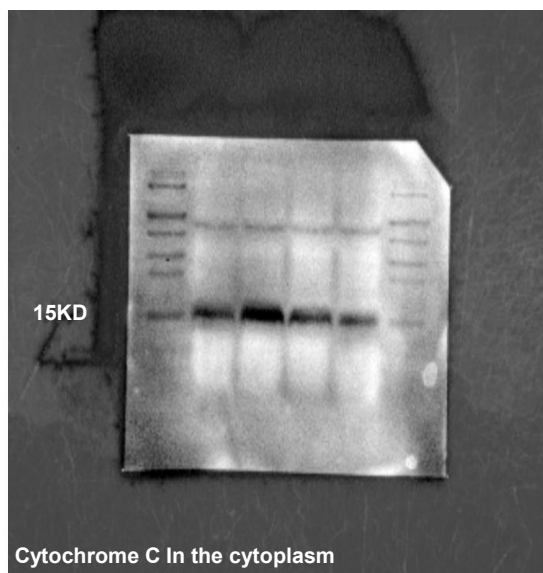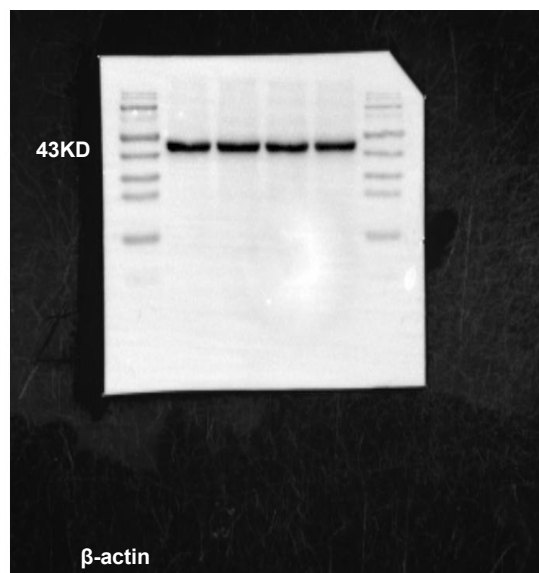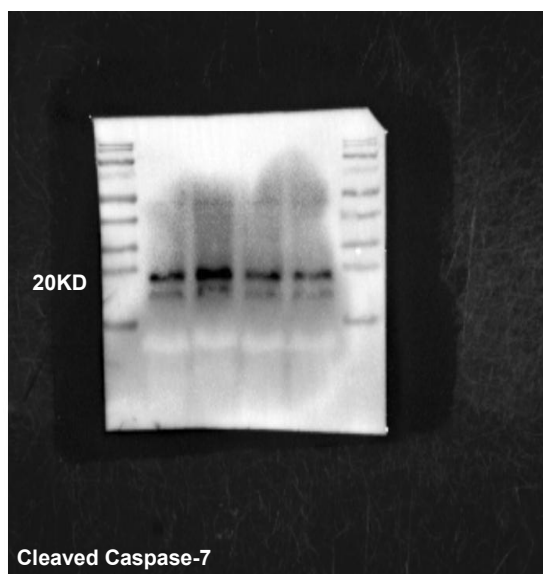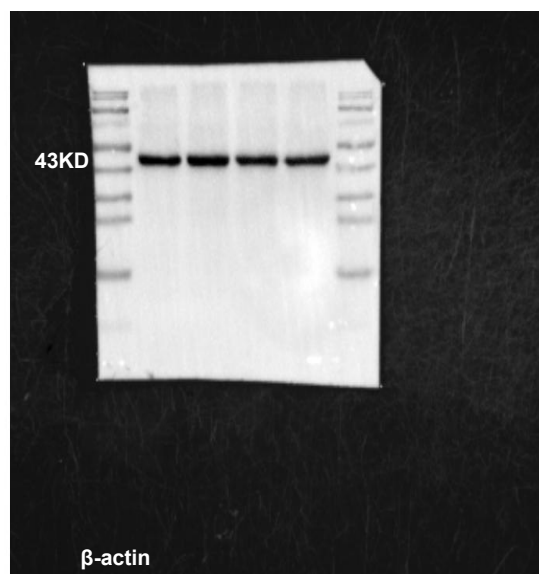

Supplement: Supplementary file 1 — Supplementary Material 1 [file 12950_2024_383_MOESM1_ESM.pdf]
